# Minor Visual Phenomena in Lewy Body Disease: A Systematic Review

**DOI:** 10.3390/biomedicines13051152

**Published:** 2025-05-09

**Authors:** Elettra Capogna, Virginia Pollarini, Alessia Quinzi, Lucia Guidi, Luisa Sambati, Maria Sasca Criante, Elena Mengoli, Annalena Venneri, Raffaele Lodi, Caterina Tonon, Micaela Mitolo

**Affiliations:** 1 Department of Biomedical and Neuromotor Sciences (DIBINEM), University of Bologna, 40127 Bologna, Italy; raffaele.lodi@unibo.it (R.L.); caterina.tonon@unibo.it (C.T.); 2 Functional and Molecular Neuroimaging Unit, IRCCS Istituto delle Scienze Neurologiche di Bologna, 40139 Bologna, Italy; virginia.pollarini3@unibo.it (V.P.); alessia.quinzi@unibo.it (A.Q.); lucia.guidi@isnb.it (L.G.); micaela.mitolo@unipr.it (M.M.); 3 U.O.C. Clinica Neurologica Rete Metropolitana (NeuroMet), IRCCS Istituto delle Scienze Neurologiche di Bologna, 40139 Bologna, Italy; luisa.sambati@isnb.it; 4 IRCCS Istituto delle Scienze Neurologiche di Bologna, 40139 Bologna, Italy; mariasasca.criante@ausl.bologna.it (M.S.C.); elena.mengoli@ausl.bologna.it (E.M.); 5 Department of Life Sciences, Brunel University London, Kingston Lane, Uxbridge UB8 3PH, UK; annalena.venneri@unipr.it; 6 Department of Medicine and Surgery, University of Parma, 43126 Parma, Italy

**Keywords:** Lewy body disease, visual illusions, pareidolias, visuoperceptual deficits, visual hallucinations, minor visual phenomena

## Abstract

Minor visual phenomena (MVP), such as visual illusions, pareidolias, feeling of presence, and passage hallucinations, are often experienced by patients with Lewy Body Disease (LBD), in addition to complex visual hallucinations (VH), even in the early stages of the disease. This systematic review aimed to provide an up-to-date literature review of the occurrence and prevalence of MVP in LBD and to assess their potential associations both with VH and visuoperceptual and visuospatial deficits. A systematic literature search was carried out in PubMed, Web of Science, APA PsycInfo, Scopus, and Cochrane Library, and a total of 44 articles were included. The included studies showed significant variability in the occurrence of MVP in the LBD population and in the assessment methods used, such as standardized scales (e.g., the noise pareidolia test), semi-structured interviews (e.g., the North-East Visual Hallucinations Interview), and clinical descriptions. Similarly to VH, MVP appears to be highly specific to LBD, helping in differential diagnosis from Alzheimer’s Disease. The overall relationship between MVP, VH, and visuoperceptual/visuospatial deficits remains unclear. Some studies found that MVP (especially pareidolic responses and presence of hallucinations) was positively correlated with VH, yet it is challenging to determine whether MVP can be considered a precursor of future VH development. Negative associations were reported between MVP (especially pareidolias) and visuoperceptual/visuospatial abilities. However, it is not clear whether these deficits serve as independent, exclusive factors in MVP occurrence or if they interact with VH as a contributing component. Gaining insight into the occurrence of these phenomena could prove beneficial for differential diagnosis, prognosis, and prediction of treatment outcomes in patients with LBD.

## 1. Introduction

Lewy Body Disease (LBD) is the second most prevalent neurodegenerative condition in older adults, after Alzheimer’s disease (AD) [1], with a higher disease burden for both patients and caregivers [2,3]. “LBD” is an umbrella term, that encompasses both dementia with Lewy bodies (DLB) and Parkinson’s disease dementia (PDD), that are often considered different aspects of the same disease spectrum [4,5], since they share the underlying neuropathology (alpha-synuclein accumulation and Lewy body aggregation), and present with similarities in their clinical manifestation. The most recent clinical criteria for the diagnosis of DLB [6] include, as core symptoms, recurrent visual hallucinations (VH), cognitive fluctuations, rapid eye movement sleep behavior disorder (RBD), and extrapyramidal (motor) symptoms within the context of a progressive decline in cognition and in daily living activities. Indicative biomarkers include reduced dopamine transporter uptake in the basal ganglia on SPECT, low 123 iodine-MIBG myocardial uptake, and polysomnographic confirmation of REM sleep without atonia. A probable diagnosis of DLB requires the presence of either two or more core clinical features or one core feature combined with at least one indicative biomarker.

The PDD criteria [7] include insidious and slowly progressive cognitive deterioration in more than one domain, affecting daily functioning, occurring in the context of established Parkinson’s disease. Clinical features of PDD include behavioral changes, such as apathy, changes in personality and mood, hallucinations, delusions, and excessive daytime sleepiness. PDD can be differentiated from DLB using the one-year rule that stipulates that DLB can be diagnosed when cognitive and neuropsychiatric symptoms appear approximately one year before the onset of motor symptoms. In both conditions, attention, executive functions, and visuospatial processing are the domains most affected in the early stages, whereas memory decline, particularly the free recall component, may occur later in the disease progression.

Visual hallucinations among the symptoms previously described can be considered one of the most frequent and debilitating for both patients and caregivers and are associated with poor outcomes [8]. Despite efforts to establish a unified framework for explaining VH [9], their precise underlying mechanisms remain unclear. Furthermore, pharmacological treatments targeting VH are often ineffective in this population [10]. In LBD, VH manifest as recurrent, vivid, well-formed images of humans or animals [6], and, by definition, are not triggered by real visual input [11,12]. In DLB patients, VH may also appear in the early stages of the disease [13], whereas its onset is usually delayed in PDD patients [14]. In addition to complex visual hallucinations, LBD patients also experience minor visual phenomena (MVP) [6,15], documented even in the early phases across the whole LBD spectrum [16]. To our knowledge, there is a lack of standardized terminology for minor visual phenomena, resulting in inconsistent usage of terms. This inconsistency manifests in two ways: different terms may be used to describe the same phenomenon, or a single term may be applied to various distinct phenomena. For example, the concept of “feeling the presence of a person nearby that cannot be seen or touched” may be referred to as “presence hallucination” [17,18], “feeling of presence” [19], “sensed presence” [20], “phantom boarder” [21]. The term “misperception” is sometimes used as an umbrella term that includes various minor phenomena [22] and is sometimes used synonymously with illusions [23]. Similarly, “pareidolias” and “metamorphopsias” are occasionally considered as distinct concepts and other times categorized as subtypes of “illusions” [20]. The absence of clear definitions underscores the importance of establishing a uniform terminology. Here, we have chosen to use “minor visual phenomena” as a comprehensive term. The most frequently used terms and studied/described phenomena encompass visual illusions, pareidolias, misperceptions, presence hallucinations, and passage hallucinations. We have selected the following definitions for these concepts. Visual illusions can be defined as misperceptions of real visual stimuli that differ from their actual appearance or nature [18,24,25], pareidolias are also described as visual illusions in which meaningful objects are perceived within ambiguous forms present in actual visual contexts [12,26,27], and misperceptions are defined as misidentification of percepts [23]. Additionally, the feeling that someone is nearby or has just left the room is referred to as presence hallucinations [28], and passage hallucinations involve the visual experience of a shadow-like figure or a person, object, or animal moving swiftly in one’s peripheral vision [29].

The relationship between MVP and VH in the progression of LBD has not been completely clarified. Indeed, it is unclear whether these minor phenomena occur alongside VH [6,20,30] or precede them [31]. To date, some studies have reported an association between VH and MVP [12,26,32], although this has not been consistently replicated [17,33]. Investigating these MVP could offer insights into their temporal trajectories and potential predictive roles in the future manifestation of VH. Moreover, some of these phenomena (i.e., pareidolias) that may be elicited in experimental conditions through standardized tools have been shown to be objective and reliable proxies of VH [12,26], and can yield additional information. Indeed, currently VH identification depends solely on clinical interviews, patient self-reports, or caregiver reports [34,35]. Consequently, this requires either patients’ insight into their symptoms or information from a reliable informant. Hence, understanding minor visual phenomena in LBD is essential from diagnostic, prognostic, and therapeutic perspectives, as it may provide more reliable information for potentially detecting VH earlier. Incorporating a standardized assessment of MVP in clinical practice could be beneficial for the initial assessment and follow-up of patients within the LBD spectrum who do not initially exhibit visual hallucinations. This approach could facilitate closer monitoring of at-risk individuals, ensure timely interventions, and possibly enhance patient outcomes.

The association between minor visual phenomena and visuoperceptual and visuospatial impairment is unclear as well, particularly for illusions and pareidolias [12,32,36]. Both symptoms frequently occur together and generally worsen over the course of the disease. Some researchers have argued that these phenomena, such as visual illusions, may arise only partially from visuospatial and visuoperceptual impairments, similar to those observed in visual hallucinations [37,38]. In contrast, others [33] urge caution when interpreting, for example, the results of a pareidolia test, emphasizing that these might not be minor visual phenomena but rather represent significant visuoperceptual deficits.

In conclusion, the underlying mechanisms driving MVP still need to be fully elucidated. It is unclear whether these phenomena share behavioral similarities and underlying neural mechanisms with VH, whether they can occur independently, are prodromal, or are merely associated with VH. Additionally, it is uncertain whether MVP arise specifically from deficits in visual processing, separate from VH, or whether they manifest in the presence of both VH and impairment in visuoperceptual and visuospatial functions. In this regard, it may be relevant to gain insights into the potential overlap of neurobiological correlates associated with MVP, VH, and impairments in visuoperceptual and visuospatial functions.

Therefore, the aim of the present systematic review was to provide an up-to-date literature review of the occurrence and prevalence of MVP in LBD, elucidating their potential associations with other clinical features, such as VH and visuoperceptual and visuospatial deficits, typically experienced by LBD patients.

## 2. Materials and Methods

### 2.1. Search Strategy

The review was carried out following the Preferred Reporting Items for Systematic Review and Meta-Analysis (PRISMA) guidelines [39]. A systematic literature search was carried out independently by four authors (EC, VP, AQ, and LG) in December 2024 using five databases (PubMed, Web of Science, APA PsycInfo, Scopus, and Cochrane Library). The following search terms were used: “Lewy Body Disease” or “Lewy Body disorders” or “dementia with Lewy bodies” or “DLB” or “LBD” combined with “illusions” or “hallucinations” or “misperceptions” or “pareidolia” or “pareidolic illusion” or “pareidolic experience” or “minor visual phenomena” or “minor visual hallucinations” or “MVH” or “passage hallucinations” or “presence hallucinations” or “subclinical hallucinations”. See Supplementary Materials for details about the search strategy. In addition, the reference lists of the selected original articles/reviews on similar topics were searched for additional eligible records.

### 2.2. Study Eligibility Criteria

Studies were considered eligible if they reported MVP, such as pareidolias, illusions, misperceptions, minor visual hallucinations, passage hallucinations, and presence hallucinations in LBD patients, including DLB and PDD, as well as patients in the prodromal stage of DLB. The following exclusion criteria were defined to identify all relevant studies: (1) no data on the target sample (2) no description or data regarding the phenomenon of interest; (3) review, meta-analysis, trials, editorial, letter articles; (4) non-peer reviewed articles; (5) articles written not in English. The year of publication was not considered as an inclusion/exclusion criterion, as we aimed to capture all existing research in this field.

### 2.3. Study Selection

The initial literature search produced a total of 1593 records, of which 534 were duplicate publications that were found in the five databases. Out of the remaining 1059 articles, 846 articles were screened based on their title and abstract according to our selection criteria. Four independent reviewers (EC, VP, AQ, and LG) performed the study selection separately to guarantee consistency of the results. Each assessor reviewed the search output to process each entry and either exclude or retain it. Any discrepancies were resolved through discussion, requiring a consensus of three out of four assessors for inclusion. A total of 213 full-text reports were retrieved and assessed for eligibility. Based on the inclusion/exclusion criteria, 132 articles did not include data or describe the phenomenon of interest; 20 reported data from meta-analyses, reviews, letters, and editorials; 13 did not include our sample of interest; 2 were written not in English; and 2 were excluded for other reasons. These latter two cases involved an educational case scenario and a study lacking information on the prevalence, number, or statistics of the phenomenon of interest within the sample.

**Figure 1:**
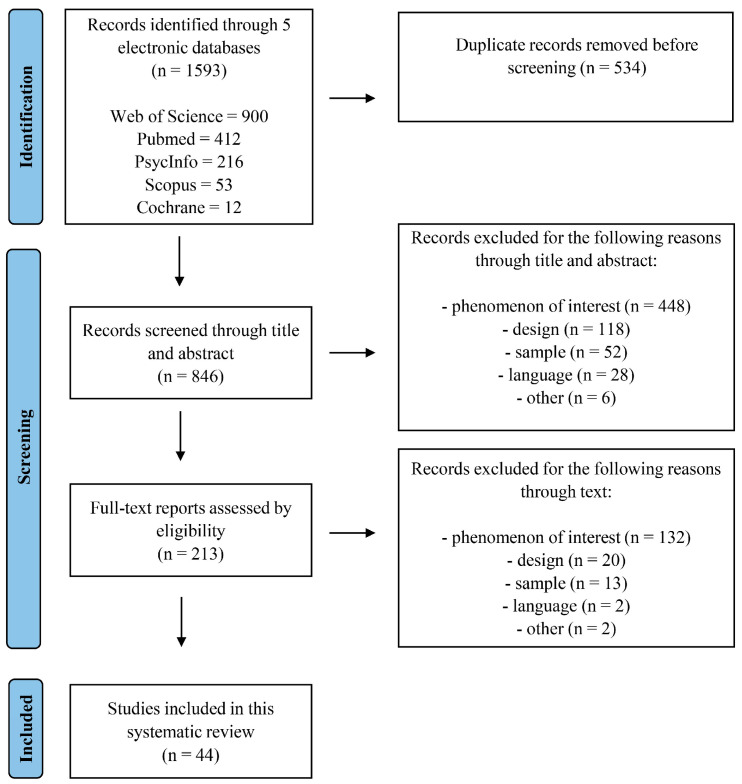
PRISMA flowchart describing the study selection process.

Therefore, 44 unique studies were included in this systematic review. The study selection process is described in Figure 1.

### 2.4. Quality Assessement

The quality of the studies included was also assessed independently by two reviewers (EC and AQ) using standardized tools: the “CARE criteria checklist” [40] for case reports and the “Effective Public Health Practice Project Quality Assessment Tool for Quantitative Studies” (EPHPP) [41] for all the other quantitative papers, following the component (A–F) ratings that contribute to the global rating for each paper.

## 3. Results

### 3.1. Study Characteristics

All details about the demographic features, the phenomenology of minor visual phenomena, the methods used for their assessment, related statistics and/or prevalence, and the main findings of each study are summarized in Table 1. All the studies included in this systematic review were published between 1996 and 2024.

The total sample encompassed 1800 participants with LBD and 221 participants with mild cognitive impairment (MCI) with Lewy Bodies. The types of phenomena investigated were pareidolias (n = 18 studies), illusions (n = 18), presence hallucinations (n = 15), passage hallucinations (n = 8), misperceptions (n = 4), with some studies exploring multiple phenomena simultaneously. Assessment methods included validated tests, such as the noise pareidolia (n = 15), the scene pareidolia test (n = 4), Bistable Percept Paradigm (n = 2), semi-structured interviews, such as the North-East Visual Hallucinations Interview (NEVHI) [34] (n = 4), Columbia University Scale for Psychopathology in Alzheimer’s disease (CUSPAD) [42] (n = 3), Questionnaire for Psychotic Experiences (QPE) [35] (n = 1), clinical descriptions, not better specified semi-structured interviews (n = 14), and medical records (n = 3), with some studies relying on multiple assessment methods simultaneously. Ten studies provided only statistics on test performance, twenty-three only the number of participants experiencing the phenomenon, nine reported both information, and two reported the number of pareidolic responses.

**Table 1:** Summary of the characteristics and main findings of the 44 studies included in this systematic review.

Authors (Year)	Sample (N)	Age Mean (sd)	Sex (F/M)	Phenomenon of Interest	MVP Assessment	Results LBD		Key Findings	Quality Index
						Statistics	Prevalence		
Alsemari and Boscarino (2024) [37]	DLB (94) MCI-DLB (97) HC (56)	70.2 (7.75) 71.11(6.98) 72.11 (8.68)	13/84 14/80 35/21	Pareidolias	Noise pareidolia test (20 items)	3.16 (3.85) 1.51 (2.32)	-	DLB patients exhibited more pareidolias than the HC and MCI groups. Visuospatial and visuoperceptual processes partially, but not independently, contribute to explaining noise pareidolia test scores. VH did not provide additional explanation for the variance in performance.	Strong (1)
Chiba et al. (2015) [43]	DLB-AD^+^ (12) DLB-AD^−^ (11) AD (10) HC (11)	77.0 (4.3) 73.0 (5.1) 74.3 (6.5) 74.6 (4.7)	3/9 5/6 8/2 6/5	FOP	Worksheet for patients and caregivers	-	11 (91.7%) 3 (27.2%)	The DLB-AD+ group (with greater parietal/precuneus hypometabolism) showed higher prevalence of VH, FOP, and higher scores on the Bender-Gestalt test, compared with DLB-AD- (lower parietal/precuneus hypometabolism).	Weak (3)
D’Antonio et al. (2022) [15]	35 LBD (19 DLB;16 PDD)	76.7 (6.5)	12/23	MVH (illusions, FOP, feeling of passage)	NEVHI	4.7 (8.2) severity 1.4 (1.8) duration 2.6 (3.02) frequency	Illusions: 9 (25.71%) FOP: 9 (25.71%) Passage: 12 (34.28%)	Distinct neuropsychological and functional network patterns underlie CVH and MVH. MVH were not associated with impaired visuoperceptual processing. MVH were negatively correlated with reduced FC in the left areas of the ventral visual stream, as well as between the brainstem and primary visual cortex.	Moderate (2)
D’Antonio et al. (2024) [17]	28 LBD with VH (16 DLB, 12 PDD) HC (20)	74.75 (5.76) -	10/18 -	MVH (illusions, FOP, feeling of passage)	NEVHI	5.43 (8.90) severity 1.57 (2.00) duration 2.93 (3.04) frequency	Illusions: 8 (28.57%); FOP: 8 (28.57%) Passage: 10 (35.71%)	MVH and CVH arose from distinct neural processes. MVH were not associated with gray matter loss. MVH were related to greater structural integrity of white matter pathways. A negative relationship was found between MVH severity and mean diffusivity, particularly for tracts linking dorsal and ventral attention networks with visual regions.	Moderate (2)
Ferman et al. (2013) [22]	LBD (41) AD (70) AD-amy LB (14)	70.2 (8.8) 69.1 (9.0) 68.0 (8.7)	17/24 36/34 10/4	Misperceptions	Interview	-	31 (76%)	Misperceptions and VH did not differ between groups, although these symptoms occurred earlier in LBD. The presence of LB pathology in the limbic and cortical regions has been linked to the occurrence of misperceptions in LBD patients.	Weak (3)
Firbank et al. (2024) [44]	LBD-VH^+^ (41) LBD-VH^−^ (48) HC (60)	75.8 (5.5) 74.2 (6.8) 74.7 (6.6)	8/33 7/41 17/43	Pareidolias, MVH (illusions, FOP, feeling of passage)	Noise pareidolia test (40 item), NEVHI	5.81 (5.03) 2.66 (3.83) [subgroup: 59]	FOP: 12 (57.14%) Passage: 10 (47.62%) Illusions: 7 (33.33%) Pareidolias: 3 (14.28%) [subgroup: 21]	DLB patients with VH exhibited reduced FC within the ventral attention network and from the visual to the DMN. Significant positive association between the number of correct responses on the pareidolia task and connectivity between the visual and DMN.	Moderate (2)
Galvin et al. (2021) [45]	DLB (110) AD (78) HC (53) MCI DLB (22) MCI AD (79)	77.7 (7.6) 79.7 (8.0) 67.6 (10.0) 75.3 (5.3) 73.5 (8.8)	30/80 43/35 37/16 7/15 38/41	Pareidolias	Noise pareidolia test (20 item)	4.0 (3.9) 1.9 (2.2)	-	DLB (and MCI/DLB) performed significantly worse on the noise pareidolia test (with different scores computed) compared with AD and HC. Utility of the pareidolia score in discriminating between DLB and AD (and MCI-DLB and MCI-AD).	Weak (3)
Hamilton et al. (2021) [46]	probable MCI-LB (43) possible MCI-LB (20) MCI-AD (40) HC (34)	74.9 (6.36) 74.1 (7.95) 76.2 (7.54) 74.2 (7.45)	7/36 9/11 23/17 10/24	Pareidolias	Noise pareidolia test (40 item)	2 [0, 14] 2 [0, 20]	-	Higher rates of pareidolias were observed in MCI-LB patients. Weak association between pareidolias and VH. The noise pareidolia test had reduced predictive value for classifying LB or VH in patients with MCI.	Weak (3)
Heitz et al. (2015) [47]	DLB-VH^+^ (36) DLB-VH^−^ (30)	71.7 (10.2) 73.5 (6.9)	14/19 10/18	Illusions	Interview	-	8 (22.22%)	Hypoperfusion in anterior and posterior brain regions was associated with VH. The occurrence of visual illusions may be specifically linked to the reduction in CBF in the cuneus. Significant association between CVH and hypoperfusion in temporal and frontal areas.	Moderate (2)
Hely et al. (1996) [48]	DLB (9)	62 (4.5)	2/7	Illusions	Clinical description	-	1 (11%)	Visual illusions were reported by one patient. VH emerged 2.5–9 years after symptom onset in six patients and were an initial manifestation in one case. Five individuals initially presented with symptoms resembling those of idiopathic PD and subsequently developed dementia.	Case report
Inagawa et al. (2020) [49]	probable DLB (24) AD (22)	82.4 (5.0) 80.0 (5.6)	14/10 11/11	Pareidolias	Noise pareidolia test (40 item)	10.6 (11.7)	-	Combined together, the pareidolia test (rate), odor stick identification test (OSIT-J), DaT-SPECT, and MIBG. Significant statistical differences were observed between DLB and AD groups, with DLB patients exhibiting higher rates of pareidolic illusions and more severe hyposmia. The pareidolia test and OSIT-J were useful in differentiating DLB from AD. The ROC curves did not show high sensitivity and specificity (compared to MIBG and DaT-SPECT).	Moderate (2)
Iseki et al. (2002) [20]	probable DLB (8)	74.5 (3.94)	2/6	Illusions, FOP	Clinical description		Illusions: 4 (50%) Presence: 4 (50%)	Patients with DLB experienced psychiatric symptoms similar to those caused by levodopa (these symptoms manifested before any medication was administered). Hallucinations and cognitive decline occurred first compared with other symptoms (such as depression and delusions). Visual illusions and feelings of presence were characteristic of DLB primarily due to visual misidentification.	Case report
Ishimaru et al. (2024) [50]	DLB (2)	83.5 (7.78)	2/0	Illusions	PA-LE, interview	-	2 (100%)	Some VH were induced by specific visual illusions (misidentification of common household objects). An individualized non-pharmacological strategy was developed for each patient by eliminating environmental triggers and improving the occurrence of VH.	Case report
Mamiya et al. (2016) [12]	probable DLB (52) AD (52) HC (20)	79.5 (7.2) 79.8 (6.2) 78.8 (5.0)	31/21 39/13 15/5	Pareidolias	Noise pareidolia test (32 items), Scene pareidolia test (10 items)	7.3 (8.4) 3.9 (1.9) 11.1 (8.6) * * Pareidolia score as the sum of images from the scene and the noise version	31 scored above the cut-off (both in the scene and noise pareidolia test)	DLB exhibited higher pareidolia responses and worse performance on the visuospatial and visuoperceptual tests. A significant correlation was observed between the scene pareidolia test score and a visuospatial component of the ACE-R. The pareidolia score (combination of scene and noise pareidolia tests) significantly correlated with clinical VH and demonstrated excellent inter-rater/test–retest reliability and better sensitivity and specificity.	Weak (3)
Matar et al. (2020) [51]	DLB (27) HC (25)	73 (63–86) 73 (55–88) median	31/21 7/18	Misperceptions	Interview, MDS-UPDRS, SCOPA-PC	-	4 (14.80%)	Factor analysis revealed six factors accounting for 81% of the total symptom variance. Misperceptions were independent of VH. VH were prominently represented in the first factor, explaining 18% of the variance along with cognitive fluctuations. The fifth factor encompassed misperceptions, apathy, and delusions.	Moderate (2)
McCann et al. (2023) [33]	DLB-PDD (13) PD (13) AD (12) PCA (5) HC (32)	75.7 (5.4) 67.5 (8.2) 66.8 (6.5) 67.8 (4.5) 67.3 (7.1)	3/10 5/8 6/6 1/4 15/17	Pareidolias	Noise pareidolia test (40 item)	7.1 (9.4)	-	No significant correlation between pareidolic responses and history of VH in LBD. LBD patients who had experienced VH showed more visuoperceptual deficits than those without VH. Across all patient groups, impaired visuoperception, rather than VH, was predictive of pareidolic responses. The PCA group showed the highest prevalence of pareidolic responses.	Moderate (2)
Morenas-Rodríguez et al. (2018) [52]	probable DLB (81)	79.8 (5.5)	48/33	FOP and feeling of passage	Structured questionnaire and retrospective review of medical records	-	FOP: 20 (25.3%) Passage: 24 (30.4%)	Cluster analysis of symptom presentation during prodromal phases of the disease. This study identified a neuropsychiatric cluster characterized by VH as the initial early symptom. In this group, patients were older, and their hallucinations were subsequently accompanied by misidentification, passage, and presence hallucinations, which emerged earlier than in clusters I and III.	Weak (3)
Mori et al. (2000) [53]	probable DLB (24) probable AD (48)	74.0 (5.8) 74.0 (7.2)	11/13 22/26	FOP	Interview	-	19 (79%)	DLB performed worse on simple and complex visuoperceptual tasks than AD. These deficits in visual perception may play a role in the onset of visual-related symptoms (VH and illusions).	Weak (3)
Moylett et al. (2019) [54]	DLB (251)	78.8 (7.7)	129/122	Illusions	Medical records	-	1 (0.2%)	Among the earliest reported complaints, memory loss and VH were the most prevalent. A wide range of other symptoms occurred, albeit less frequently, including those aligned with DLB criteria, such as hallucinations and related phenomena (e.g., illusions).	Weak (3)
Nagahama et al. (2007) [30]	DLB (100)	77.2 (6.5)	69/31	FOP	Semi-structured interview (caregivers, patients)	-	23 (23%)	Factor analysis of the relationships among psychotic symptoms. The study revealed that feelings of presence and VH were clustered together. This factor accounted for 8.2% of the variance in the data.	Weak (3)
Nakata et al. (2022) [55]	DLB (147) MCI DLB (15)	78.9 (6.1)	91/56	Pareidolias	Noise pareidolia test (40 item)	7.1 (7)	90 (61.22%) Cut off ≥ 3	Weak correlation between the noise pareidolia scores and CBF in frontal, cingulate gyrus, and left parietal cortex (supramarginal gyrus). No correlation with the CBF of occipital regions was reported. Pareidolias might also be affected by attentional deficits.	Weak (3)
Nicastro et al. (2020) [19]	DLB (25)	71.9 (6.7)	8/17	FOP	Medical records	-	9 (36%)	Subjects with FOP showed hypometabolism in left frontoparietal areas, including the superior parietal lobule and precuneus. The presence of VH was not associated with FOP.	Weak (3)
Oishi et al. (2020) [56]	DLB (37) AD (58) HC (32)	81.2 (6.7) 80.2 (5.9) 79.4 (4.1)	26/11 42/16 16/16	Pareidolias	Noise pareidolia test (40 items), object-identifying test	- 2.0 (2.4)	29 (78.3%) (object identification test). 23 pareidolias (noise), 21 pareidolia-like responses (object identifying test)	Visual texture agnosia was associated with the impairment of object recognition and visual misidentification (pareidolias) in DLB. Pareidolia-like responses were likely to occur when patients could not use texture as a supporting cue with ambiguous shape information for object recognition.	Weak (3)
Phillips et al. (2021) [57]	DLB (23) HC (20)	73.91 69.95		Misperceptions	Bistable percept paradigm (BPP), PsycH-Q	5.83 misperceptions	-	DLB showed more misperceptions and misses than HC. Significant correlation between the Visual Misperception subscale of PsycH-Q and the number of misperceptions in BPP. DLB with VH had more misperceptions.	Moderate (2)
Phillips et al. (2022) [23]	iRBD-MCI probable prodromal DLB (12) DLB (1) iRBD-CN (34)	70.5 (7.7) 84 69.3 (7.4)	0/1	Misperceptions	BPP	1.25 (1.3) [Only 4 completed the BPP]	-	Group differences between iRBD-CN (19) and iRBD-MCI (4) in the BPP (for misses, no misperceptions or error rate). No differences on SART and mental rotation tests. One subject who converted to DLB had 11 misses (and no misperceptions) in the BPP test at baseline.	Weak (3)
Posner et al. (2001) [58]	DLB (1)	64	0/1	Illusions	Clinical description	-	1 (100%)	Development of visual symptoms such as visual illusions and parkinsonism occurred very early in the course of the disease, along with progressive cognitive impairment (e.g., in visuospatial skills).	Case report
Rahman-Filipiak et al. (2022) [59]	LBD (56) AD (44) aMCI (96) non-aMCI (61) HC (202)	72.49 (6.75) 75.86 (8.43) 74.35 (8.84) 71.02 (7.35) 72.52 (6.88)	8/48 20/24 45/51 31/30 142/60	Pareidolias	Noise pareidolia task (20 items) (NACC Version- short version with faces)	3.44 (4.04)	-	LBD (especially those with VH) performed worse on the noise pareidolia task (on correct faces and in pareidolic errors). VH might represent misperceptions of real visual stimuli due to poor visual integration. The speeded attention and noise pareidolia task showed good convergent and discriminant validity, hence showing promising clinical utility.	Weak (3)
Reckner et al. (2020) [32]	25 LBD with FOP (7 probable DLB, 18 PD) PD without FOP (25)	67.0 (7.1) 67.7 (5.2) 64.4 (5.7)	1/6 4/14 9/16	FOP	Semi-structured interview	-	FOP: 7 (100%) Passage: 4 (57.1%)	FOP may occur in DLB and PD patients. Patients with FOP showed more impairment in visual processing skills, more visual hallucinations, and feeling of passage phenomena.	Moderate (2)
Revankar et al. (2024) [60]	DLB (25) AD (29) PD (5) HC (11)	73.7 (5.5) 73.4 (6.4) 66.6 (11.3) 62.1 (9.5)	6/19 18/11 1/4 3/8	Pareidolias	Noise pareidolia test digital and paper (40 items)	8.1 (10.3)	35 (DLB + AD + PD)	Pareidolias were captured on both versions of the pareidolia noise test (paper and digital). DLB patients had more pareidolias compared with the other groups, especially in those experiencing VH.	Weak (3)
Rothenberg et al. (2023) [61]	DLB (4)	70 (8.28)	0/4	Illusions	Clinical description	-	2 (50%)	Case description of 4 male DLB patients with visual illusions, hallucinations, and paranoid delusions. Typical medications prescribed for DLB with psychosis did not improve the symptoms; however, only pimavanserin seemed to be useful and tolerable by patients.	Case report
Stavitsky et al. (2006) [62]	DLB (28) AD (55)	73.46 (7.56) 73.09 (8.26)	9/19 34/21	Illusions	CUSPAD	-	8 (32%) [subgroup: 25 patients]	At baseline, DLB patients experienced more visual illusions, VH, and impairment in the visuoconstructional domain than AD patients. These symptoms tend to be relatively stable in DLB over the course of the disease, whereas they appear later in AD patients.	Moderate (2)
Suárez-González et al. (2014) [25]	probable DLB (80) probable AD (85)	75.9 (13.0) 74.0 (7.0)	36/44 55/30	Illusions, FOP	CUSPAD	-	Illusions: 10 (12.5%) FOP: 18 (22.5%)	DLB patients showed significantly more VH, visual illusions, and feeling of presence than AD patients. Illusions were observed only in DLB patients.	Weak (3)
Sumi et al. (2022) [18]	DLB (3) [converted from iRBD (36)]	76 (1.73)	1/2	FOP, feeling of passage, illusions, pareidolias	Semi-structured interview, noise pairedolia test (32 items)	-	Baseline: Illusions: 2 (66.66%), FOP: 2 (66.66%), Passage: 1 (33.33%) Follow-up: Illusions: 2 (66.66%), FOP: 3 (100%), Passage: 1 (33.33%) Pareidolias not reported	Two iRBD patients who initially experienced MVP progressed to DLB during follow-up and developed more severe VH. Another patient who did not initially report minor visual phenomena later experienced a feeling of presence. The progression rate was notably higher in individuals with minor visual phenomena.	Strong (1)
Suzuki et al. (2017) [63]	DLB (8) HC (9)	77.4 (6.9) 71.4 (6.7)	5/3 1/8	Pareidolias	Scene pareidolia test (25 items)	-	123 pareidolias responses	Changes in pupil diameter were observed in DLB patients prior to the occurrence of pareidolias.	Moderate (2)
Taomoto et al. (2022) [64]	prodromal DLB (2)	76 (9.90)	1/1	Illusions	Clinical description	-	1 (50%)	Two years before the onset of delusional infestation, a patient with prodromal DLB (with SAH sequelae) began complaining of visual illusions, mistaking lint for insects.	Case report
Taomoto et al. (2024) [65]	prodromal DLB (5)	64 (8)	2/3	Illusion, feeling of passage, FOP	Clinical description	-	Illusions: 1 (20%) Passage: 1 (20%), FOP: 1 (20%)	Two of the five patients with delirium-onset prodromal DLB experienced (among other symptoms) visual illusions, and feeling of passage months before the onset of delirium.	Case report
Uchiyama et al. (2012) [27]	probable DLB (34) AD (34) HC (26)	81.0 (3.9) 80.0 (3.6) 79.2 (4.9)	19/15 10/24 8/18	Pareidolias	Scene pareidolia test (25 item)	15.5 (median) 11.0 (IQR)	-	DLB patients displayed a higher number of pareidolia responses than AD patients and HC. Positive association between scene pareidolia scores and VH only in those who did not take donepezil. Negative relationship between scene pareidolia scores and face recognition tasks in participants using donepezil.	Moderate (2)
Urwyler et al. (2016) [66]	LBD (79) * PDD (48); DLB (31) ED (135), PD (156) HC (164)	74.8 (7.4) 79.8 (8.3) 70.9 (9.4) 72.9 (8.2)	23/56 93/42 64/92 92/72	Illusions, FOP/feeling of passage	NEVHI	-	Illusions: 34 (43%) FOP/passage: 52 (66%)	LBD patients primarily experienced complex VH, illusions, and feeling of passage/FOP compared with PD and ED. ED patients predominantly encountered simple VH. Simple VH may be associated with pathology in the primary retinocortical pathway, and complex VH were likely associated with higher-level cortical dysfunction in the context of LB pathology.	Moderate (2)
van de Beek et al. (2021) [67]	DLB (100) probable DLB (73) MCI (27)	69 (6) 70 (5) 67 (7)	10/90 8/65 2/25	MVH (illusions, feeling of passage, FOP)	QPE	-	Ilusions: 27 (27%), Passage: 25 (25%), FOP: 23 (23%)	VH were less prevalent, occurring in less than 50% of patients. Some patients also experienced illusions, feelings of passage, and presence. Discrepancy between NPI and QPE assessments regarding the presence of VH.	Moderate (2)
Watanabe et al. (2018) [68]	DLB (36) AD (12)	79.8 (7.4) 78.8 (6.2)	22/14 10/2	Pareidolias	Noise pareidolia test (80 item)	11.2 (16.1)	27 (75%) at least one pareidolia, 24 (66.66%) at least two pareidolias	Negative mood led to a twofold increase in pareidolic illusions among DLB patients compared to neutral mood. AD patients exhibited no notable differences in pareidolic responses between negative and neutral emotional conditions.	Moderate (2)
Watanabe et al. (2023) [36]	DLB (43)	78.3 (5.6)	26/17	Pareidolias	Scene pareidolia test (25 item)	13.5 (7.6)	43 (100%)	Pareidolia responses contributed to both VH and visual processing impairment. Hypoperfusion in the occipitotemporal and posterior parietal regions was correlated with higher scores on Factor 1 (hallucinations) and lower scores on Factor 3 (visual processing).	Weak (3)
Watanabe et al. (2020) [69]	probable DLB (1)	71	1/0	Pareidolias	Noise pareidolia test (32 item)		1 (100%) with 3 pareidolic responses	4.5 years after the onset of language impairment, a patient with PPA exhibited pareidolic responses, visual hallucinations, and additional symptoms associated with DLB diagnosis. These observations indicated the potential existence of a prodromal DLB phase characterized by PPA.	Case report
Yokoi et al. (2014) [26]	probable DLB (34) AD (34) HC (28)	79.4 (0.9) 77.7 (0.8) 78.0 (0.5)	21/13 25/9 16/12	Pareidolias	Noise pareidolia test (40 item object and 40 item face), Scene pareidolia test (25 item) [subgroup: 11 patients]	12.6 (3.3) object, 15.4 (3.2) face	-	The noise pareidolia test (face version) demonstrated a stronger association with VH and better differentiation between DLB and AD groups than the object version. DLB exhibited more illusory responses than those with AD and HC. Significant positive relationship between pareidolias and VH. Significant negative association between VH and visuospatial tests (shape detection and spatial span assessments).	Moderate (2)
Yoshizawa et al. (2013) [70]	pure DLB (12) DLB + AD (23) pure AD (89)	68.5 (6.2) 66.0 (8.6) 68.3 (9.8)	3/9 8/15 47/42	Illusions	CUSPAD		33.3% illusions 5% illusions [subgroups: 9 pure DLB and 20 DLB + AD patients ]	Patients with pure DLB experienced a higher occurrence of visual illusions and VH at the initial assessment than those with pure AD. Patients with pure DLB demonstrated greater impairment in visuospatial functions but less severe memory recognition deficits than those with pure AD and DLB + AD.	Moderate (2)

ACE-R—Addenbrooke’s Cognitive Examination Revised, AD—Alzheimer’s Disease, AD-amy LB—Alzheimer’s Disease with amygdala-predominant Lewy bodies, aMCI—amnestic mild cognitive impairment, BPP—Bistable Percept Paradigm, CBF—cerebral blood flow, CN—cognitively normal, CVH—complex visual hallucinations, CUSPAD—Columbia University Scale for Psychopathology in Alzheimer’s Disease, DaT-SPECT—dopamine transporter single photon emission computed tomography, DLB—Dementia with Lewy Bodies, DMN—Default Mode Network, ED—eye disease, FC—functional connectivity, FOP—feeling of presence, HC—healthy controls, iRBD—idiopathic rapid-eye-movement sleep behavior disorder, LB—Lewy Bodies, LBD—Lewy Body Disease, MCI—mild cognitive impairment, MDS-UPDRS—unified Parkinson’s disease rating scale, MIBG—Iodine-123 metaiodobenzylguanidine, MMSE—Mini-Mental State Examination, MVP—minor visual phenomena, MVH—minor visual hallucinations including only illusions, feeling of passage and presence, NACC—National Alzheimer’s Coordinating Center, NEVHI—North-East Visual Hallucinations Interview, NPI—Neuropsychiatric Inventory, PA-LE—Photo Assessment of Living Environment, PCA—Posterior cortical atrophy, PD—Parkinson’s Disease, PDD—Parkinson’s Disease Dementia, PLS—partial least squares, PPA—Primary progressive aphasia, PsycH-Q—Psychosis and Hallucinations Questionnaire, QPE—Questionnaire for Psychotic Experiences, SAH—subarachnoid hemorrhage, SART—Sustained Attention Response Task, SCOPA-PC—SCales for Outcomes in PArkinson’s disease-Psychiatric Complications, VH—visual hallucinations.

Differences in how results are presented are largely attributed to the variety of assessment tools employed. For instance, the noise pareidolia test yields data on performance, whereas clinical interviews offer a qualitative analysis of the phenomenon, detailing aspects such as modality, duration, frequency, and severity. This heterogeneity in assessment tools may influence the reported prevalence rates.

### 3.2. Minor Visual Phenomena

#### 3.2.1. Pareidolias

The most investigated phenomena were pareidolias (n = 18 studies) and illusions (n = 18 studies). In terms of pareidolias, seven studies reported the prevalence of the phenomenon in LBD patients. These studies found varying percentages of patients experiencing pareidolias ranging from 14% [44] to 100% [36]. Specifically, several authors outlined a prevalence of approximately 60–70% [12,55,68] among DLB patients who scored higher on the noise pareidolia test. The majority of pareidolia responses involved people and animals [26], and this finding aligns with the contents observed in VH in LBD. Additionally, pareidolia-like responses were observed in an object identification test (prevalence 78.3%) [56] and in the digital version of the noise pareidolia test [60].

Furthermore, the majority of studies examining pareidolias employed validated and structured tools (e.g., the scene and noise pareidolia tests), hence presenting findings in terms of relative statistics, comparing different samples. DLB patients exhibited more pareidolic responses compared with AD patients and healthy controls (HC) [12,26,37,45,49,59,60]. One study also noted 123 pareidolic responses within a sample of 9 DLB patients [63]. The scene and noise (especially the face version) pareidolia tests seem to be reliable in discriminating between DLB and AD patients. This distinction is also observable in the prodromal stage, with several studies reporting higher rates of pareidolias in MCI-DLB than in MCI-AD [45,46]. Another study found that negative mood may lead to a two-fold increase in pareidolic illusions only among DLB patients compared with neutral mood, whereas no differences were observed in AD patients [68]. However, only one study reported that patients with posterior cortical atrophy (PCA) exhibited the highest prevalence of pareidolic responses compared with AD and Parkinson’s disease (PD) [33], with no statistically significant differences reported between LBD patients and the other patient groups.

#### 3.2.2. Illusions and Misperceptions

Regarding illusions, most of the studies reported the prevalence of the phenomenon in LBD samples, relying on semi-structured interviews such as the NEVHI, CUSPAD, and QPE. Four studies [15,17,44,66] used the NEVHI and found that 26–43% of patients experienced illusions. Three studies [25,62,70] used the CUSPAD and compared LBD with AD patients. Stavitsky et al. [62] reported that, in early stages, 32% of DLB patients experienced more visual illusions than AD patients, illusions that tended to remain relatively stable in DLB over the course of the disease, whereas they appeared later in AD patients. Another study [25] observed illusions only in DLB patients (12.5% prevalence). Yoshizawa et al. [70] compared pure DLB, pure AD, and DLB with concomitant AD pathology (DLB + AD), as determined by *post-mortem* examination, and reported a higher occurrence of illusions (33.3%) in pure DLB cases when compared with pure AD. However, this increased prevalence was not observed when comparing pure DLB and DLB + AD cases. Moreover, only one study [67] using the QPE found visual illusions in 27% of DLB patients. In the prodromal phases of the disease, one longitudinal study [18] assessing patients with isolated RBD showed that out of the three patients who converted to DLB over time, two initially experienced illusions. Conversely, however, a retrospective study [54] examining the medical records of future DLB patients found that only 0.2% reported visual illusions as early complaints, with VH, memory loss, and depression being the most prevalent symptoms.

Regarding misperceptions, we found only four studies, with two using interviews [22,51] and the other two using the Bistable Percept Paradigm [23,57]. Ferman et al. [22] indicated that 76% of LBD patients experienced misperceptions according to an informant interview. These misperceptions occurred earlier in the disease course, compared with patients with AD and AD with amygdala predominant Lewy Bodies (AD-amy LB); however, misperceptions and VH did not differentiate LBD from AD and AD-amy LB. Another study [51], conducted through patient interviews, found a 14.80% prevalence of misperceptions. Phillips used the Bistable Percept Paradigm and found that DLB showed more misperceptions compared with HC [57]. Nevertheless, no significant differences were observed in MCI (RBD-MCI) compared with the HC group [23], with one patient converting to DLB and another one to PD.

#### 3.2.3. Presence Hallucinations and Passage Hallucinations

Several studies (n = 15) have investigated presence hallucinations, also referred to as feeling of presence, which is one of the most assessed minor visual phenomena after pareidolias and illusions. Among them, seven studies [2,15,17,32,44,52,66] also evaluated passage hallucinations, also referred to as feeling of passage. These phenomena were identified through different semi-structured interviews, showing globally that 23–100% of LBD patients experienced feeling of presence and 25–57.1% reported feeling of passage. Studies [15,17,44,66] using the NEVHI as an assessment method found a prevalence ranging from 28% to 57% of patients experiencing feeling of presence, and between 34% and 48% of patients experiencing feeling of passage. Among these, Urwyler et al. [66] reported a combined prevalence of 66% for both phenomena in LBD patients that was higher than in other patient groups (i.e., patients with Eye Disease and PD). Another study [67], using the QPE, observed similar prevalence rates in DLB patients: 23% for the feeling of presence and 25% for the feeling of passage. Suárez-González et al. [25], focusing only on the feeling of presence using the CUSPAD, found that DLB patients showed significantly more feeling of presence phenomena compared with AD patients. Conversely, another study [43] reported that DLB patients who presented with AD pathology showed a higher prevalence of feeling of presence compared with DLB patients who did not. Other authors [32,52] used various methods (structured questionnaire, medical records, semi-structured interview) to assess both phenomena, observing that between 25.3% and 100% of patients experienced feeling of presence, while 34.4–57.1% reported feeling of passage. As for the phenomenology of feeling of presence, Nicastro et al. [19] observed that 33% of patients with feeling of presence perceived the experience from behind, while 66.7% sensed it from the side with no particular side preference. These patients also demonstrated more feeling of passage phenomena [32]. In the early stages of the disease, three isolated RBD patients who progressed to DLB had feeling of presence, and one reported feeling of passage during follow-up [18].

### 3.3. Associations Between Minor Visual Phenomena and Visual Hallucinations

A comprehensive overview of the findings is presented in Supplementary Table S1. All studies, except one, investigated complex VH relying on different tools. Assessment methods included the Neuropsychiatric Inventory (NPI) [71] (n = 16), NEVHI (n = 5), CUSPAD (n = 3), MDS-UPDRS (n = 3), psycH-Q (n = 2), QPE (n = 1), and SCOPA-PC (n = 1). Additionally, some studies used unspecified interviews (n = 12), clinical description and medical records (n = 13), with some studies using multiple assessments simultaneously.

Out of all studies, seventeen conducted statistical analyses to explore the relationship between minor visual phenomena and visual hallucinations. Seventeen other studies merely described the presence of both phenomena without formal testing [18,20,25,43,44,48,50,55,57,61,62,64,65,66,67,70]. These studies used different methods, mainly focusing on pareidolias and feeling of presence.

In relation to pareidolias, seven studies [12,26,27,36,59,60,68] demonstrated a significant positive association between pareidolias, particularly when evaluated using the noise pareidolia test, and VH, primarily assessed through NPI. Consequently, DLB patients experiencing visual hallucinations exhibited more pareidolic responses than those without VH. Additionally, one study [27] reported a significant positive correlation between illusory responses and hallucinations (total, frequency, and severity) only in those not taking an acetylcholinesterase inhibitor (donepezil), whereas no significant association was found for those taking this medication. Conversely, three studies [33,37,46] failed to establish a statistically significant effect of VH on the occurrence of pareidolias. For instance, one study [46] that focused on the prodromal phases of the disease found that VH were not predictive of pareidolic response in MCI with Lewy bodies patients, reporting only a weak relationship between the two phenomena when VH were measured using the NEVHI. In this instance, the noise pareidolia test seemed to have low sensitivity in distinguishing MCI with Lewy bodies from MCI-AD and HC groups.

For feeling of presence, we found four studies that reported a statistical relationship between this phenomenon and VH [19,30,32,52]. Two studies using a data-driven approach found that the feeling of presence and hallucinations belonged to the same factor or cluster [30,52]. Contrasting results were reported in two studies that compared patients with and without feeling of presence. One study indicated that patients with feeling of presence showed more VH and feeling of passage [32], whereas another found no significant VH differences between patients with feeling of presence and without [19].

For other minor visual phenomena, several studies found no association between minor visual hallucinations (referring in this context to illusions, feeling of presence, and feeling of passage) and complex VH [15,47]. For instance, Matar et al. [51] reported that misperceptions were independent of VH. Without directly testing this association, other studies observed that VH and illusions occurred more frequently in DLB compared with AD patients [25,62,70] and other patient groups and HC [66].

### 3.4. Associations Between Minor Visual Phenomena and Visuoperceptual/Visuospatial Impairment

The main findings are presented in Supplementary Table S2. Fourteen studies assessed the statistical association between minor visual phenomena and visuoperceptual/spatial abilities, whereas three merely described the occurrence of MVP and possible visuoperceptual/spatial impairment within the same subjects. Among the fourteen studies cited above, nine reported statistically significant results, with all focusing on pareidolias. Only one study focused on feeling of presence and found that patients with feeling of presence showed more frequent impairments in visual processing than those without feeling of presence [32]. Most studies [12,26,33,59] reported a significant negative correlation between the pareidolia test score and visuospatial scores, as measured by various neuropsychological tests such as the Visual Object and Space Perception Battery (VOSP) subtests [72], the Benson Complex Figure Test [73], and the ACE-R subtest [74]. For instance, Yokoi et al. [26] found significant negative correlations between illusory responses on the noise pareidolia test and shape detection, face recognition, and spatial span in a DLB sample. Uchiyama et al. [27], using the scene pareidolia test, reported negative correlations between illusory responses and face recognition, but only in DLB patients taking donepezil. Additionally, another study [56] noted a significant negative correlation between the number of pareidolia-like responses and visual texture agnosia, as measured using a material identification test. Two studies [36,37] used different methodological approaches, specifically factor analysis and multiple regression analysis, demonstrating that scores on visual processing tests explain the occurrence of pareidolia responses. According to McCann [33], the characteristic visuoperceptual deficits in LBD patients were thought to influence their performance on pareidolia tests, potentially skewing the interpretation of results. Five studies [15,47,52,53,57] failed to find a statistically significant association between MVP and visuoperceptual/visuospatial test performance, respectively, for illusions and misperceptions using the Rey-Osterrieth Complex Figure (ROCF) test [47,57], and for feeling of presence using the ROCF and Number localization subtest-VOSP [52], and a subset of visual perceptual tests [53]. Without directly testing this association, three other studies observed that pure DLB exhibited higher visual illusions and greater visuospatial and visuoconstructional impairments compared with AD [62,70], whereas Chiba et al. [43] found that DLB-AD+ patients also experienced a greater feeling of presence phenomena, compared with DLB-AD-, AD, and HC and had higher scores on the Bender-Gestalt test, indicating poorer performance.

Most of the studies examined in this review did not allow us to distinguish between patients’ visuoperceptual and visuospatial abilities. Only three studies [26,27,56] reported significant correlations between pareidolias and visuoperceptual deficits, whereas no such relationship was observed for visuospatial impairments.

### 3.5. Neural Correlates Underlying Minor Visual Phenomena

Nine studies investigated the neural correlates underlying minor visual phenomena using various methods. For pareidolias, two studies focused on cerebral blood flow measured using SPECT, yielding results that were not fully consistent [36,55]. Indeed, one study [55] found a weak correlation between the noise pareidolia score and cerebral blood flow in frontal, cingulate gyrus, and left parietal regions, independently of VH, and no association with occipital regions. Conversely, Watanabe et al. [36] identified a relationship between pareidolia responses, VH, and visual processing impairment and reported that pareidolic illusions may arise from hypoperfusion in occipitotemporal, frontal, and perisylvian regions. Another study [44] using resting state functional magnetic resonance imaging found significant positive associations between the number of correct responses on the pareidolia task and connectivity between the visual and default mode networks, while connectivity within the ventral attention network was correlated with visuospatial performance. Additionally, changes in pupil diameter were observed in DLB patients prior to the occurrence of pareidolias, possibly resulting from decreased arousal levels [63].

For other minor visual phenomena, two studies examined feeling of presence [19,43] using PET. Nicastro et al. [19] reported that DLB with feeling of presence showed hypometabolism in left frontoparietal regions. Another study found that DLB patients with higher parietal/precuneus hypometabolism that resembled a typical AD pattern experienced more feeling of presence phenomena, compared with DLB without AD pathology [43]. Heitz et al. [47] outlined that visual illusions may be specifically associated with reduced blood flow in the cuneus. Furthermore, minor VH (referring in this context to illusions, feeling of presence, and feeling of passage) and complex VH may be underpinned by different functional and structural network patterns [15,17]. Indeed, minor VH severity was correlated with reduced functional connectivity within regions of the ventral visual stream and between the brainstem and primary visual regions [15]. Moreover, minor VH were not associated with gray matter loss; instead, they are related to greater structural white matter integrity, especially for tracts linking dorsal and ventral attention networks with visual regions [17].

### 3.6. Case Report

Eight studies were found: six case series and two case reports that documented the following phenomena: illusions (n = 7 studies), feeling of presence (n = 2), passage (n = 1), and pareidolias (n = 1). Two studies [20,65] explored multiple phenomena simultaneously. Watanabe et al. [69] detailed a patient with PPA attributed to DLB, who, after the onset of language impairment, exhibited pareidolic responses, VH, and other DLB-related symptoms. At follow-up, hallucinations persisted, pareidolic responses decreased, and visuospatial abilities remained intact across timepoints. Another case report [58] described a DLB patient with preserved insights into his own difficulties, who developed visual illusions (not VH) and parkinsonism in the early course of the disease, accompanied by progressive cognitive decline, including visuospatial deficits. All case series [20,48,50,61,64,65] reported patients who experienced visual illusions and VH. Two studies [50,61] explored potentially beneficial treatments (non-pharmacological and pharmacological) for symptom improvement. Two studies [64,65] focused on delirium-onset in prodromal DLB patients and reported that this symptom is sometimes preceded by minor visual phenomena such as visual illusions and feeling of passage. Finally, Iseki et al. [20] described DLB patients experiencing visual illusions and feeling of presence, combined with VH of people, and occasionally animals, which appeared characteristic of LBD, primarily due to visual misidentification.

### 3.7. Quality Assessment Results

All quantitative studies (n = 36) were evaluated using the EPHPP. Of these, sixteen studies reported a moderate global rating, three were rated strong, and seventeen were classified as weak. See Supplementary Table S3 for additional details. Given the nature of the studies, some biases were clearly present: randomization or blinding techniques were either not feasible or not documented; researchers were typically aware of group compositions; and confounding factors were not always fully controlled. However, the data collection methods were generally robust, with 25 studies achieving a strong rating, based on the validity and reliability of the instruments used. The case reports (n = 8) were evaluated according to the CARE checklist, and all of them provided comprehensive information on patients, clinical findings, and timeline information. See Supplementary Table S4.

## 4. Discussion

This systematic review includes 44 studies, each examining at least one minor visual phenomenon of interest, with a primary focus on pareidolias, illusions, and presence hallucinations. Our aim was to gain insight into the occurrence and prevalence of these phenomena in the LBD population. We also sought to identify the potential stages in which these symptoms manifest throughout the course of the disease and whether they are specific features of LBD, along with other symptoms such as visual hallucinations and visuoperceptual/visuospatial impairment.

Overall, the included studies showed significant variability in the occurrence of minor visual phenomena in the LBD population, with some studies reporting high prevalence rates, whereas others showed lower percentages for the same phenomenon. Furthermore, assessment methods differed considerably across studies, with most studies relying on clinical interviews, except for pareidolias that were primarily investigated using validated tests. Consequently, the presentation of findings varies widely across studies, ranging from simple descriptions of the presence/absence of the phenomena to comprehensive statistical analyses.

Similar to VH, these phenomena appear to be highly specific to LBD, allowing for the differential diagnosis from other neurological groups, such as AD [27,45]. Indeed, LBD patients experienced more pareidolic responses, illusions, and feelings of presence compared with AD and HC groups [25,37,62]. For instance, the pareidolia score [12] obtained from the noise [26] and scene pareidolia test [27] could discriminate DLB from AD. Specifically, the scene test showed good discriminatory ability but had weak correlation with clinical visual hallucinations, whereas the noise test, particularly its face version, correlated strongly with visual hallucinations but was less specific in differentiating DLB from AD compared with the scene pareidolia test. The noise and scene pareidolia tests showed a strong correlation with each other.

Furthermore, it is pivotal to understand whether minor visual phenomena arise from, are independent of, or are associated with visual hallucinations. Studies in our review that investigated the relationship between these phenomena and VH yielded conflicting results, making it challenging to determine whether these symptoms can be considered VH proxies or not. This inconsistency highlights the need for further research to clarify these associations and their underlying mechanisms. Indeed, while some studies reported positive associations between pareidolias and VH [12,26,27,36,59,60,68], others failed to establish a significant association [33,37,46]. Similarly, for feeling of presence, a few studies observed that patients with feeling of presence experienced more VH [32], whereas another did not report the same association [19]. VH and pareidolias share similar phenomenological features, for example, in their content, and some authors [26,47] explained these phenomena through the lens of the Perception and Attention Deficit model [38], a framework that has been proposed for explaining visual hallucinations. It has also been suggested that while pareidolias and VH may be influenced by bottom-up processing, an impairment of top-down processes might play a crucial and specific role in the mechanisms underlying pareidolias compared with VH. Indeed, a negative association has been reported between pareidolic responses and atrophy in the thalamus and occipitotemporal regions that are involved in bottom-up processing, as well as the orbitofrontal cortex that plays a role in top-down processing [75]. Hence, it is possible that the MVP in LBD patients results from a lack of integration between top-down and bottom-up mechanisms. This has also been suggested by a recent study in drug-naïve patients with PD, especially for pareidolias [75]. Moreover, impaired connectivity within the ventral attention network and between visual regions and the default mode network could contribute to impaired performance in the pareidolia task [44]. Other studies identified lower hypoperfusion in occipital regions [36,47] underlying the occurrence of visual illusions and pareidolic responses. However, other authors have suggested that minor VH (illusions, feeling of presence, and feeling of passage) and complex VH are underpinned by different neuropsychological, functional, and structural network patterns [15,17]. Indeed, minor VH severity was correlated with reduced functional connectivity within regions of the ventral visual stream and between the brainstem and primary visual regions [15]; in particular, illusions may arise from functional connectivity alterations in the lateral occipital cortex, whereas passage hallucinations may be related to altered functional connectivity in the parahippocampal regions due to its role in visuospatial processing.

Other studies have examined the association between minor visual phenomena and visuoperceptual/visuospatial impairment, commonly experienced by LBD patients. However, these investigations have not yielded clear conclusions. All studies reported statistically significant findings focused on pareidolias, with the exception of the feeling of presence [32]. These findings suggest that higher pareidolia responses were negatively correlated with lower performance on visual processing and visuospatial test scores [12,26,33]. Some authors [12] suggested that while visuoperceptual deficits contribute to pareidolias, they are not sufficient to cause them. In contrast, others [33] proposed that pareidolias may be due only to visuoperceptual impairment, independently of VH. This perspective emphasizes the limitations of using tests or items with strong visual-perceptual components when investigating MVP. This hypothesis is supported by the observation that a small PCA group, characterized by visual perception deficits without VH, demonstrated a particularly high occurrence of increased pareidolic responses compared with the other groups. The majority of studies examining other minor visual phenomena [15,17,47,52,53,57] failed to find a statistically significant association between minor VH (especially for illusions and misperceptions) and visuoperceptual/spatial test performance. For some authors [15], minor VH (illusions, feeling of presence, and feeling of passage) and complex VH are underpinned by different neuropsychological profiles. Minor VH, which likely precede complex VH, were not associated with cognitive impairment. In contrast, complex visual hallucinations were associated with deficits in visuoperceptual processing and visual attention, supporting the hypothesis of impairment in the ventral visual stream.

As for the neurobiological underpinnings of MVP, studies included in this review found that pareidolias were associated with hypoperfusion in frontoparietal and occipitotemporal regions [36,55]. Reduced connectivity within the ventral visual stream (occipitotemporal regions) was associated with illusions, feelings of presence, and passage [15,36]. Based on these findings, we can speculate that visuoperceptual impairment, as proposed by the Perception and Attention Deficits Model [38], and visuospatial/attentional control deficits, associated with default mode network engagement, as described by the Attentional Network Dysfunction model [76,77], may play a role in the development of MVP. Overall, the neural correlates underlying MVP, visuoperceptual/visuospatial impairment, and VH observed in LBD patients seem to be partially overlapping and involve occipitotemporal regions. Indeed, it is well documented that posterior hypometabolism is a supportive criterion for the diagnosis of DLB [6] and is related to impairment of visuoperceptual and visuospatial functions [78]. On the other hand, VH appear to be related to increased activity in areas within the visual ventral and dorsal stream that are normally involved during perception of real external stimuli, for instance, the fusiform face area (occipitotemporal regions) is functionally active during hallucinations involving faces [79].

Most of the studies included documented medication use among patients. Many of these patients are treated with medications acting on the dopaminergic system, such as levodopa, carbidopa, premipexole, and MAO-B inhibitors, forms of treatment that have been hypothesized to contribute to the onset of hallucinations [80,81]. However, the studies included in this review that investigated the influence of these treatments have generally failed to demonstrate a significant impact of antiparkinsonian drugs on either minor or major hallucinations [22,30,32]. To manage psychotic symptoms, atypical antipsychotics, including quetiapine, risperidone, and pimavanserin, are the most commonly prescribed drugs. These agents have shown benefits in some reported cases despite the risk of exacerbating motor symptoms [82]. Some studies have suggested a possible association between cholinergic deficiency and the development of MVP [26,27,69]. Indeed, some patients showed improvement in these symptoms after taking an acetylcholinesterase inhibitor (AChEI), such as donepezil, rivastigmine, or galantamine. However, it remains unclear whether acetylcholine plays a primary role in MVP or if its effects are secondary, resulting from enhanced cognitive functions, such as attention and visuoperceptual abilities [33]. One study showed that DLB patients experienced significantly more visual hallucinations, illusions, and feelings of presence than AD patients, with all AD participants taking AChEIs [25]. However, another study that compared DLB and AD patients found no statistically significant differences in AChEI dosage between groups, despite DLB patients having more pareidolic responses and poorer visuospatial/visuoperceptual performance [12]. Several of the studies mentioned have found no significant differences in medication use between patients experiencing visual hallucinations and those who did not [25,26]. Additionally, no significant correlations were observed between minor VH or complex VH severity and concurrent medication use [15,17]. Case reports have provided partially conflicting results. Indeed, one patient reported improvements in language and global cognitive functions after the administration of donepezil, as well as a reduction in pareidolias, but not in visual hallucinations [69]. Another study indicated that pimavanserin improved hallucinations, illusions, and paranoid delusions in four male patients with DLB and appeared more tolerable than first- and second-generation antipsychotics [61]. Overall, further research is needed to investigate the effects of these medications on MVP, VH, and visuoperception/visuospatial impairment [60].

Nevertheless, to investigate whether minor visual manifestations precede other symptoms, it would be necessary to focus on LBD patients in the early stages of the disorder. In our review of the literature, we found only one study [46] that examined the prodromal phases of the disease, with the aim of evaluating the utility of the pareidolia test in differentiating MCI-DLB from MCI-AD. MCI-DLB had more pareidolic responses than the MCI-AD and HC groups. However, the correlation between these responses and visual hallucinations was minimal, with the authors highlighting that their sample experienced fewer than average hallucinations. Moreover, a ROC analysis indicated that, although the pareidolia test had good specificity, it showed low sensitivity in discriminating DLB from AD in the early stages of disease. Other studies focusing on MVP in patients with idiopathic REM behavior disorder (iRBD), a population at high risk of developing LBD, have shown significant associations between MVP and several variables [18,23,83,84]. These included cognitive decline, the onset of visual hallucinations, and a higher conversion rate to LBD. These findings underscore the potential predictive value of MVP in this specific patient group. Consequently, more longitudinal studies with bigger samples, also encompassing patients in the prodromal stages of the disease, are needed to understand the progression of these phenomena and to clarify whether they occur in the earlier phases of the course of the disease. Gaining insight into the occurrence of these phenomena could have a role in differential diagnosis, prognosis, and prediction of treatment outcomes in patients with LBD.

The current literature is not exempt from limitations. First, there is a large inconsistency in the terminology used to describe these phenomena, making it more difficult to understand whether different terms are referring to the same phenomena (e.g., visual illusions, pareidolias, and misperceptions). The heterogeneity in terminology might stem not only from a lack of consensus among researchers but also from the variability in clinical manifestations that often partially overlap and lack clearly defined boundaries. The phenomenology of hallucinations can be viewed as a continuum [85,86,87], with complex visual hallucinations at one end, gradually decreasing in intensity to simple visual hallucinations, such as colored dots, lights, or patterns. We have chosen not to categorize complex and simple visual hallucinations under the broad term of MVP, as they are distinctly identified as occurring without external stimuli, belonging to the hallucinatory extreme. At the opposite end, there is a spectrum of minor perceptual phenomena, primarily visual (MVP), and these are the focus of this review and are triggered by a real stimulus [6,15]. Additionally, we consider that phenomena such as presence hallucinations and passage hallucinations can be placed in the middle of this continuum due to their ambiguous nature that combines misperception and hallucinatory features. These are more akin to minor visual phenomena, and thus, we have included them in our classification. Second, another limitation is the heterogeneity of assessment methodologies used in the studies included in this review, with some using qualitative interviews, as well as in the presentation of results, with some merely indicating the presence or absence of the phenomena. Moreover, some studies have investigated multiple phenomena concurrently and have reported their occurrence. However, some studies failed to clarify whether the same subjects experienced more phenomena simultaneously or whether different subjects presented with different phenomena. This might hamper the generalizability of the study findings. Hence, more quantitative studies using validated and standardized assessment tools are needed. This approach could improve the consistency of the findings, thus facilitating the calculation of prevalence rates and of the overall combined effect size (through a meta-analysis) of the phenomena of interest. Furthermore, testing of inter-rater reliability would have strengthened the methodological rigor of our systematic review.

Lastly, the lack of longitudinal studies in LBD and of those that have tested patients in the early phases of the disease hinders our understanding of the trajectories of minor visual phenomena over time and their associations with visuoperceptual/visuospatial impairment and visual hallucinations.

## 5. Conclusions

In conclusion, this systematic review elucidated the occurrence and prevalence of minor visual phenomena in LBD patients and explored their potential associations with visual hallucinations and visuoperceptual/visuospatial deficits commonly experienced by these patients. The variability in results and the tools used for assessment make it challenging to determine a general prevalence pattern among these patients; this may lead to an underestimation of these phenomena, especially for pareidolias. Pareidolias, illusions, and feelings of presence were the most frequently studied and observed phenomena specifically in LBD patients compared with other patient groups. Although they differ in their phenomenological characteristics, they often manifest in the same patient at different times or simultaneously, and thus these symptoms can be collectively referred to as minor visual phenomena. Understanding these phenomena could be valuable for improving differential diagnosis and predicting disease progression. Further research, particularly longitudinal studies with larger samples including patients in the early stages of disease, is necessary to tackle the progression and nature of these phenomena.

## Data Availability

The original contributions presented in this study are included in the article/Supplementary Materials. Further inquiries can be directed to the corresponding author.

## Supplementary Materials

The following supporting information can be downloaded at: https://www.mdpi.com/article/10.3390/biomedicines13051152/s1, Search strategy, Figure S1. Number of studies assessing and reporting MVP; Figure S2. Weighted histogram of phenomena by percentage, Supplementary Table S1. Summary of the characteristics, visual hallucinations assessment, and statistical associations between minor visual phenomena and visual hallucinations of the 44 studies included in the systematic review; Supplementary Table S2. Summary of the characteristics, neuropsychological assessment, and statistical associations between minor visual phenomena and visuoperceptual/visuospatial abilities of the 44 studies included in the systematic review; Supplementary Table S3. Quality assessment for all quantitative studies (n = 36) using the Effective Public Health Practice Project Quality Assessment Tool for Quantitative Studies (EPHPP); Supplementary Table S4. Quality assessment for all case reports (n = 7) using the “CARE criteria checklist”.

## Abbreviations

The following abbreviations are used in this manuscript:

LBD	Lewy body disease
AD	Alzheimer’s disease
DLB	Dementia with Lewy bodies
PDD	Parkinson’s disease dementia
VH	Visual hallucinations
RBD	Rapid eye movement sleep behavior disorder
MVP	Minor visual phenomena
MCI	Mild cognitive impairment
NEVHI	North-East Visual Hallucinations Interview
CUSPAD	Columbia University Scale for Psychopathology in Alzheimer’s disease
QPE	Questionnaire for Psychotic Experiences
PD	Parkinson’s disease
NPI	Neuropsychiatric Inventory
