# Supplementary material for: Minor Visual Phenomena in Lewy Body Disease: A Systematic Review
Source: Biomedicines. 2025 May 9;13(5):1152. doi: 10.3390/biomedicines13051152 (PMC12108890; doi:10.3390/biomedicines13051152)
Supplement: Supplementary file 1 [file biomedicines-13-01152-s001.zip › biomedicines-3557776-supplementary.pdf]

Search strategy

Date: 09/12/2024

Databases: Web of Science, Pubmed, APA PsycInfo, Cochrane, Scopus

Total: 1593

Web of Science

(ALL=("lewy body disease") OR ALL=("lewy body disorders") OR ALL=("dementia with lewy bodies") OR ALL=("DLB") OR ALL=("LBD")) AND (ALL=("illusions") OR ALL=("hallucinations") OR ALL=("misperception") OR ALL=("pareidolia") OR ALL=("pareidolic illusions") OR ALL=("pareidolic experience") OR ALL=("minor visual phenomena") OR ALL=("minor hallucinations") OR ALL=("minor visual hallucinations") OR ALL=("MVH") OR ALL=("passage hallucinations") OR ALL=("presence hallucinations") OR ALL=("subclinical hallucinations"))

Results: 900

Pubmed

(((Lewy Body Disease [MesH Terms]) OR (Lewy Body disorders[All fields]) OR (dementia with lewy bodies [All fields]) OR (DLB [All fields]) OR (LBD [All fields])) AND ((illusions[Mesh Terms]) OR (hallucinations[Mesh Terms]) OR (misperception[All fields]) OR (pareidolia[All fields]) OR (pareidolic illusion[All fields]) OR (pareidolic experience[All fields]) OR (minor visual phenomena[All fields]) OR (minor visual hallucinations[All fields]) OR (minor hallucinations[All fields]) OR (MVH[All fields]) OR (passage hallucinations[All fields]) OR (presence hallucinations[All fields]) OR (subclinical hallucinations[All fields])))

Results: 412

Mesh Terms

- **Lewy Body Disease:**

- Diffuse Lewy Body Disease
- Dementia, Lewy Body
- Cortical Lewy Body Disease
- Lewy Body Disease, Cortical

- Lewy Body Type Senile Dementia
- Lewy Body Disease, Diffuse
- Lewy Body Dementia

- **Illusion**

- Autokinetic Illusions
- Autokinetic Illusion
- Illusion, Autokinetic
- Illusions, Autokinetic
- Autokinetic Effect
- Effect, Autokinetic
- Autokinetic Effects
- Effects, Autokinetic
- Illusions, Kinesthetic
- Illusion, Kinesthetic
- Kinesthetic Illusion
- Kinesthetic Illusions
- Illusions, Tactile
- Illusion, Tactile
- Tactile Illusion
- Tactile Illusions
- Illusions, Visual
- Illusion, Visual
- Visual Illusion
- Visual Illusions
- Illusions, Auditory
- Auditory Illusion
- Auditory Illusions
- Illusion, Auditory

- **Hallucinations**:

- Hallucination
- Hallucinations, Hypnagogic
- Hallucination, Hypnagogic
- Hypnagogic Hallucination
- Hypnagogic Hallucinations
- Hallucinations, Gustatory
- Gustatory Hallucination
- Gustatory Hallucinations
- Hallucination, Gustatory
- Hallucinations, Formed, of People
- Hallucinations, Elementary
- Elementary Hallucination
- Elementary Hallucinations
- Hallucination, Elementary
- Hallucinations, Dissociative
- Dissociative Hallucination
- Dissociative Hallucinations
- Hallucination, Dissociative
- Hallucinations, Auditory
- Auditory Hallucination
- Auditory Hallucinations
- Hallucination, Auditory
- Hallucination of Body Sensation
- Body Sensation Hallucination
- Body Sensation Hallucinations
- Hallucinations, Hypnapompic
- Hallucination, Hypnapompic
- Hypnapompic Hallucination
- Hypnapompic Hallucinations
- Hallucinations, Internal Body Sensation

- Hallucinations, Kinesthetic
- Hallucination, Kinesthetic
- Kinesthetic Hallucination
- Kinesthetic Hallucinations
- Hallucinations, Mood Congruent
- Hallucination, Mood Congruent
- Mood Congruent Hallucination
- Mood Congruent Hallucinations
- Hallucinations, Mood Incongruent
- Hallucination, Mood Incongruent
- Mood Incongruent Hallucination
- Mood Incongruent Hallucinations
- Hallucinations, Olfactory
- Hallucination, Olfactory
- Olfactory Hallucination
- Olfactory Hallucinations
- Hallucinations, Organic
- Hallucination, Organic
- Organic Hallucination
- Organic Hallucinations
- Hallucinations, Reflex
- Hallucination, Reflex
- Reflex Hallucination
- Reflex Hallucinations
- Hallucinations, Sensory
- Hallucination, Sensory
- Sensory Hallucination
- Sensory Hallucinations
- Hallucinations, Somatic
- Hallucination, Somatic
- Somatic Hallucination

- Somatic Hallucinations
- Hallucinations, Tactile
- Hallucination, Tactile
- Tactile Hallucination
- Tactile Hallucinations
- Hallucinations, Verbal Auditory
- Auditory Hallucinations, Verbal
- Auditory Hallucination, Verbal
- Hallucination, Verbal Auditory
- Verbal Auditory Hallucination
- Verbal Auditory Hallucinations
- Hallucinations, Visual
- Hallucination, Visual
- Visual Hallucination
- Visual Hallucinations
- Hallucinations, Visual, Formed
- Hallucinations, Visual, Unformed

APA PsycInfo

(((MA Lewy Body Disease) OR (TX lewy body disorders) OR (TX dementia with lewy bodies) OR (TX DLB) OR (TX LBD)) AND ((MA illusions) OR (MA hallucinations) OR (TX misperception) OR (TX pareidolia) OR (TX pareidolic illusion) OR (TX pareidolic experience) OR (TX minor visual phenomena) OR (TX minor visual hallucinations) OR (TX minor hallucinations) OR (TX MVH) OR (TX passage hallucinations) OR (TX presence hallucinations) OR (TX subclinical hallucinations)))

Results: 216

Scopus

((ALL(lewy body disease) AND ALL(illusions))) OR ((ALL(lewy body disorders) AND ALL(illusions))) OR ((ALL(dementia with lewy bodies) AND ALL(illusions))) OR ((ALL(DLB) AND ALL(illusions))) OR ((ALL(LBD) AND ALL(illusions))) OR ((ALL(lewy body disease) AND ALL(hallucinations))) OR ((ALL(lewy body disorders) AND ALL(hallucinations))) OR ((ALL(dementia with lewy bodies) AND ALL(hallucinations))) OR ((ALL(DLB) AND ALL(hallucinations))) OR ((ALL(LBD) AND ALL(hallucinations))) OR ((ALL(lewy body disease) AND ALL(misperception))) OR ((ALL(lewy body disease) AND ALL(pareidolia))) OR ((ALL(lewy body disease) AND ALL(pareidolic illusion))) OR ((ALL(lewy body disease) AND ALL(pareidolic experience))) ((ALL(lewy body disease) AND ALL(minor visual phenomena))) OR ((ALL(lewy body disease) AND ALL(minor hallucinations))) OR ((ALL(lewy body disease) AND ALL(minor visual hallucinations))) OR ((ALL(lewy body disease) AND ALL(MVH))) OR ((ALL(lewy body disease) AND ALL(passage hallucinations))) OR ((ALL(lewy body disease) AND ALL(presence hallucinations))) OR ((ALL(lewy body disease) AND ALL(subclinical hallucinations))) OR ((ALL(lewy body disorders) AND ALL(misperception))) OR ((ALL(lewy body disorders) AND ALL(pareidolia))) OR ((ALL(lewy body disorders) AND ALL(pareidolic illusion))) OR ((ALL(lewy body disorders) AND ALL(pareidolic experience))) ((ALL(lewy body disorders) AND ALL(minor visual phenomena))) OR ((ALL(lewy body disorders) AND ALL(minor hallucinations))) OR ((ALL(lewy body disorders) AND ALL(minor visual hallucinations))) OR ((ALL(lewy body disorders) AND ALL(MVH))) OR ((ALL(lewy body disorders) AND ALL(passage hallucinations))) OR ((ALL(lewy body disorders) AND ALL(presence hallucinations))) OR ((ALL(lewy body disorders) AND ALL(subclinical hallucinations))) OR ((ALL(dementia with lewy bodies) AND ALL(misperception))) OR ((ALL(dementia with lewy bodies) AND ALL(pareidolia))) OR ((ALL(dementia with lewy bodies) AND ALL(pareidolic illusion))) OR ((ALL(dementia with lewy bodies) AND ALL(pareidolic experience))) ((ALL(dementia with lewy bodies) AND ALL(minor visual phenomena))) OR ((ALL(dementia with lewy bodies) AND ALL(minor hallucinations))) OR ((ALL(dementia with lewy bodies) AND ALL(minor visual hallucinations))) OR ((ALL(dementia with lewy bodies) AND ALL(MVH))) OR ((ALL(dementia with lewy bodies) AND ALL(passage hallucinations))) OR ((ALL(dementia with lewy bodies) AND ALL(presence hallucinations)))

OR ((ALL(dementia with lewy bodies) AND ALL(subclinical hallucinations))) OR ((ALL(DLB) AND ALL(misperception))) OR ((ALL(DLB) AND ALL(pareidolia))) OR ((ALL(DLB) AND ALL(pareidolic illusion))) OR ((ALL(DLB) AND ALL(pareidolic experience))) ((ALL(DLB) AND ALL(minor visual phenomena))) OR ((ALL (DLB) AND ALL(minor hallucinations))) OR ((ALL(DLB) AND ALL(minor visual hallucinations))) OR ((ALL(DLB) AND ALL(MVH))) OR ((ALL(DLB) AND ALL(passage hallucinations))) OR ((ALL(DLB) AND ALL(presence hallucinations))) OR ((ALL(DLB) AND ALL(subclinical hallucinations))) OR ((ALL(LBD) AND ALL(misperception))) OR ((ALL(LBD) AND ALL(pareidolia))) OR ((ALL(LBD) AND ALL(pareidolic illusion))) OR ((ALL(LBD) AND ALL(pareidolic experience))) ((ALL(LBD) AND ALL(minor visual phenomena))) OR ((ALL(LBD) AND ALL(minor hallucinations))) OR ((ALL(LBD) AND ALL(minor visual hallucinations))) OR ((ALL(LBD) AND ALL(MVH))) OR ((ALL(LBD) AND ALL(passage hallucinations))) OR ((ALL(LBD) AND ALL(presence hallucinations))) OR ((ALL(LBD) AND ALL(subclinical hallucinations)))

Results: 53

Cochrane Library

#**1** MeSH descriptor: [Lewy Body Disease] explode all trees

#2 “lewy body disorders” OR “dementia with lewy bodies” OR “DLB” OR “LBD”

#**3** MeSH descriptor: [Illusions] explode all trees

#**4** MeSH descriptor: [hallucinations] explode all trees

#**5** “misperception” OR “pareidolia” OR “pareidolic illusion” OR “pareidolic experience” OR “minor visual phenomena” OR “minor visual hallucinations” OR “minor hallucinations” OR “MVH” OR “passage hallucinations” OR “presence hallucinations” OR “subclinical hallucinations”

#**6** (#1 OR #2) AND (#3 OR #4 OR #5)

Results: 12

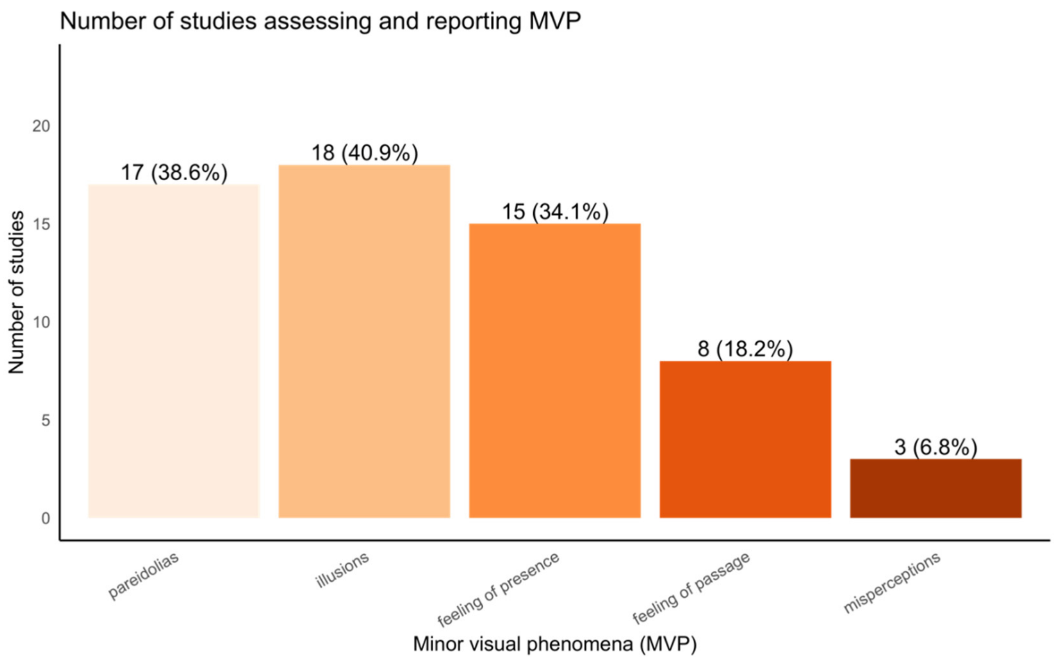

Supplementary Figure S1 Number of studies assessing and reporting MVP. The histograms reflect the number of studies investigating and reporting minor visual phenomena. The percentage is calculated based on a total of 44 studies.

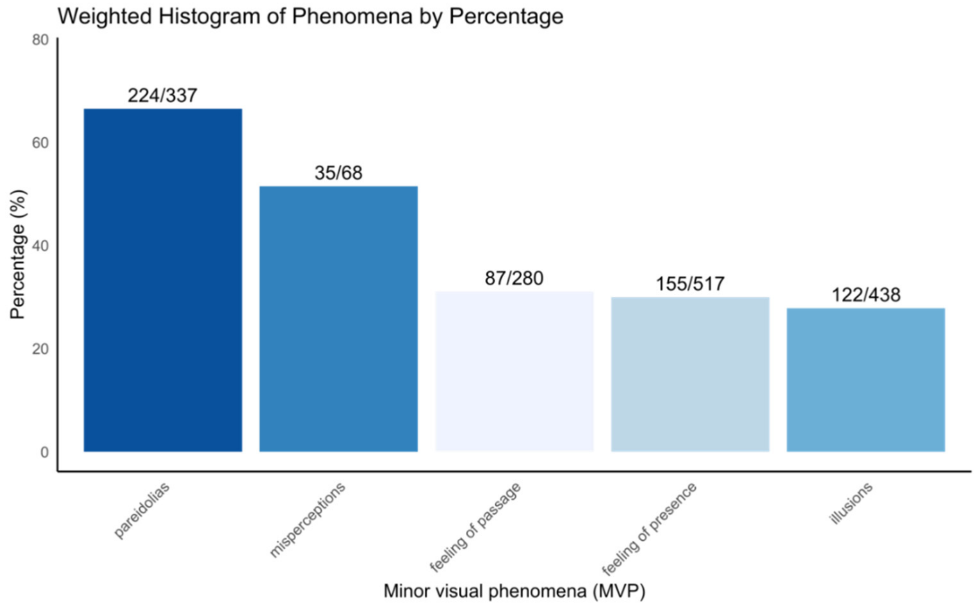

Supplementary Figure S2 Weighted histogram of phenomena by percentage. The histograms illustrate the percentage of each phenomenon, calculated by dividing the number of participants experiencing minor visual phenomena by the total number of participants tested for these phenomena. The numerical values displayed above each histogram represent these values.

Note: the plot is based on data from only 30 out of 44 studies, as the remaining 14 studies did not provide prevalence statistics.

Supplementary Table S1 Summary of the characteristics, visual hallucinations assessment, and statistical associations between minor visual phenomena and visual hallucinations of the 44 studies included in the systematic review

Authors (year)	Sample (N)	Phenomenon	VH assessment	Associations with VH	Statistical Analysis
Alsemari and Boscarino (2024) [37]	DLB (94)<br>DLB-MCI (97)<br>HC (56)	Pareidolias	NPI	VH did not significantly account for any additional variance beyond NPS performance.	Hierarchical multiple linear regression
Chiba et al. (2015) [43]	DLB-AD^{+} (12),<br>DLB-AD^{-} (11)<br>AD (10)<br>HC (11)	FOP	Worksheet for the patient and the caregiver	DLB-AD^{+} higher VH compared with HC, DLB-AD^{-}, AD<br>*no statistical association with the minor visual phenomena*	-
D'Antonio et al. (2022) [15]	35 LBD<br>(19 DLB and16 PDD)	MVH (illusions, feeling of presence, feeling of passage)	NEVHI	Not tested	-
D'Antonio et al. (2024) [17]	28 LBD with history of VH (16 DLB, 12 PDD)<br>HC (20)	MVH (illusions, feeling of presence, feeling of passage)	NEVHI	No significant association between MVH and CVH.	Pearson correlation
Ferman et al. (2013) [22]	LBD (41)<br>AD (70)<br>AD amy-LB (14)	Misperceptions	Interview	Not tested	-

Firbank et al. (2024) [44]	LBD-VH (41) LBD-noVH (48) among them DLB (32), MCI-LB (38), MCI-PD/PDD (34); HC (60)	Pareidolias, MVH (illusions, feeling of presence, feeling of passage)	NPI, NEVHI, interview	LBD-VH higher pareidolias *no statistical association with minor visual phenomena*	-
Galvin et al. (2021) [45]	DLB (110) AD (78) HC (53) MCI DLB (22) MCI AD (79)	Pareidolias	NPI	Not tested	-
Hamilton et al. (2021) [46]	probable MCI-LB (43) possible MCI-LB (20) MCI-AD (40) HC (34)	Pareidolias	NPI, NEVHI, interview	VH not predictive of pareidolic responses, weak relationship between pareidolias and the severity of VH measured by the NEVHI (no with NPI, interview). Noise pareidolia test had good specificity but low sensitivity in differentiating MCI-LB from MCI-AD and HC.	Generalised linear mixed models Pearson correlation ROC analysis
Heitz et al. (2015) [47]	DLB VH (36) DLB no VH (30)	Illusions	Interview, medical records	Subgroup comparisons based on the type of hallucinations (complex, illusions, simple) showed no significant clinical differences between the group experiencing illusions and the other two groups.	Kruskal-Wallis test

Hely et al. (1996) [48]	DLB (9)	Illusions	Clinical description	One patient with illusions had also complex VH *no statistical association with minor visual phenomena*	-
Inagawa et al. (2020) [49]	probable DLB (24) AD (22)	Pareidolias	-	Not tested	-
Iseki et al. (2002) [20]	probable DLB (8)	Illusions, FOP	Clinical description	All patients (n = 4) experiencing visual illusions also reported visual hallucinations (VH) of people, while two of these patients additionally experienced VH of animals. All subjects who encountered presence phenomena also reported VH of people, and one of them further experienced auditory hallucinations. *not a statistical association with minor visual phenomena*	-
Ishimaru et al. (2024) [50]	DLB (2)	Illusions	NPI, interview	Visual illusions triggers (from the PALE) some of the visual hallucinations *no statistical association with minor visual phenomena*	-
Mamiya et al. (2016) [12]	probable DLB (52) AD (52)	Pareidolias	NPI	Significant positive correlation between noise pareidolia test	Spearman's correlation

	HC (20)			and pareidolia score and NPI hallucination score.<br>No significant association between scene test and NPI hallucination score.	
Matar et al. (2020) [51]	DLB (27)<br>HC (25)	Misperception	Interview, MDS-UPDRS, SCOPA-PC	Misperceptions independent of VH, placed in different factors.	Exploratory factor analysis
McCann et al. (2023) [33]	DLB-PDD (13)<br>PD (13)<br>AD (12)<br>PCA (5)<br>HC (32)	Pareidolias	History of VH medical records, interview (patients, caregivers)	Non-significant trend for VH and pareidolic responses.<br>Non-significant differences in pareidolic responses between LBD hallucinators and non-hallucinators.<br>VH no significant predictor of pareidolic responses.	Spearman correlation<br>Wilcoxon signed-rank test<br>Linear regression
Morenas-Rodríguez et al. (2018) [52]	Probable DLB (81)	FOP and passage	Structured questionnaire and retrospective review of medical records	In cluster II, which was characterized by neuropsychiatric onset, visual hallucinations emerged as the initial symptoms, preceding sense of presence, and passage hallucinations. These phenomena appeared earlier in cluster II compared with cluster I (cognitive predominant) and cluster III (parkinsonism). While the prevalence of these symptoms did not differ	K-means clustering method- t-test-Mann-Whitney U test

				significantly among clusters, there were notable differences in the disease duration at the onset of these manifestations.	
Mori et al. (2020) [53]	probable DLB (24)<br>probable AD (48)	FOP	NPI	Not tested	-
Moylett et al. (2019) [54]	DLB (251)	Illusions	Medical records	Not tested	-
Nagahama et al. (2007) [30]	DLB (100)	FOP	Semi-structured interview (caregivers, patients)	FOP and hallucination of people in the same factor (that accounted for 8.2% of the variance, psychotic symptoms).	Factor analysis
Nakata et al. (2022) [55]	DLB (147)<br>Among which MCI DLB (15)	Pareidolias	Clinical data	Of the 40 patients with hallucinations, 27 were positive for pareidolia (noise pareidolia score ≥ 3). Among those without VH (107), 63 were positive for pareidolia<br>*no statistical association with minor visual phenomena*	-
Nicastro et al. (2020) [19]	DLB (25)	FOP	Medical records	No significant differences between FOP+ and FOP- in term of VH.	Chi-squared group differences
Oishi et al. (2020) [56]	DLB (37)<br>AD (58)	Pareidolias	NPI	Not tested	-

	HC (32)				
Phillips et al. (2021) [57]	DLB (23)<br>HC (20)	Misperceptions	MDS-UPDRS item 1.2, PsycH-Q	VH patients experienced more misperceptions at BPP<br>*no statistical association with minor visual phenomena*	-
Phillips et al. (2022) [23]	iRBD-MCI probable prodromal DLB (4)	Misperceptions	MDS-UPDRS item 1.2, PsycH-Q, unstructured interview for prodromal signs of visual hallucinations	Not tested	-
Posner et al. (2001) [58]	DLB (1)	Illusions	-	No tested	-
Rahman-Filipiak et al (2022) [59]	LBD (56)<br>AD (44)<br>aMCI (96)<br>non-aMCI (61)<br>HC (202)	Pareidolias	interview	VH were associated with lower scores on noise pareidolia test.	One-way ANOVA
Reckner et al. (2020) [32]	probable DLB (7)<br>PD (18)<br>*with FOP:25*<br>PD *without FOP* (25)	FOP	Interview	Patients with FOP, compared with those without FOP, showed more VH and feeling of passage.	Group comparison t-test
Revankar et al. (2024) [60]	DLB (25)<br>AD (29)<br>PD (5)<br>HC (11)	Pareidolias	Clinical description	Positive significant association between patients with VH and pareidolia score.	Chi-squared group comparison

Rothenberg et al. (2023) [61]	DLB (4)	Illusions	Clinical description	Patients with illusions also experienced VH *no statistical association with minor visual phenomena*	-
Stavitsky et al. (2006) [62]	DLB (28) AD (55)	Illusions	CUSPAD	DLB patients compared with AD showed significantly more VH and visual illusions *no statistical association with minor visual phenomena*	-
Suárez-González et al. (2014) [25]	probable DLB (80) probable AD (85)	Illusions, FOP	NPI, CUSPAD	DLB patients compared with AD showed significantly more VH, visual illusions, and feeling of presence. *no statistical association with minor visual phenomena*	-
Sumi et al. (2022) [18]	DLB (3) *converted from a sample of iRBD (36)*	FOP, feeling of passage, illusions, pareidolias	Semi-structured interview, CGI-S	Two patients with minor visual phenomena at baseline had more severe VH at follow-up *no statistical association with minor visual phenomena*	-
Suzuki et al. (2017) [63]	DLB (8) HC (9)	Pareidolias	NPI	Not tested	-
Taomoto et al. (2022) [64]	Prodromal DLB (2)	Illusions	Clinical description	One patient had visual illusions and visual hallucinations prodromal of delusion infestation	-

				no statistical association with minor visual phenomena	
Taomoto et al. (2024) [65]	Prodromal DLB (5)	Illusion, feeling of passage, FOP	Clinical description	Patients with minor visual phenomena had also VH. The one with illusions didn't experience VH anymore after delirium phase was over *no statistical association with minor visual phenomena*	-
Uchiyama et al. (2012) [27]	probable DLB (34) AD (34) HC (26)	Pareidolias	NPI, medical records	Significant positive correlation between illusory responses and hallucinations (total, frequency, severity) only in those without donepezil. Significant positive correlation between illusory responses and hallucinations (frequency) in the entire DLB group. No significant associations between illusory responses and hallucinations for those taking donepezil	Spearman's correlation
Urwyler et al. (2016) [66]	LBD (79)* ED (135), PD (156) HC (164) *among which PDD (48) and DLB (31)	Illusions, FOP and feeling of passage (combined)	NEVHI	VH phenomenology significantly predicted the disease (complex VH for LBD) FOP/passage and illusions were more common in LBD than in other groups, complex VH were mostly found in LBD patients	Logistic regression Chi-squared frequency

				no statistical association with minor visual phenomena	
van de Beek et al. (2021) [67]	Total DLB (100) Probable DLB (73) MCI (27)	MVH (illusions, passage, presence)	QPE, NPI	39 patients experienced hallucinations. We could not establish whether they are the same patients experiencing minor visual phenomena. *no statistical association with minor visual phenomena*	
Watanabe et al. (2018) [68]	DLB (36) AD (12)	Pareidolias	NPI	More VH predicted higher degrees of affective modulation of pareidolic illusions	Multiple linear regression analysis
Watanabe et al. (2023) [36]	DLB (43)	Pareidolias	NPI	Factor 1 (hallucinations/fluctuations) weighted strongly on higher hallucinations score, fluctuations score and pareidolia responses.	Factor analysis
Watanabe et al. (2020) [69]	Probable DLB (1)	Pareidolias	NPI	At baseline, the patients experienced hallucinations and exhibited pareidolic responses. At follow-up, hallucinations persisted, but the patient demonstrated a decrease in pareidolic responses. *no statistical association with minor visual phenomena*	-
Yokoi et al. (2014)	probable DLB (34)	Pareidolias	NPI	significant positive correlation between illusory responses (face	Spearman's correlation

[26]	AD (34)<br>HC (28)			version) and hallucinations (total, frequency, severity). Patients with VH showed higher illusory responses (face version) compared with patients without VH.	
Yoshizawa et al. (2013)<br>[70]	pure DLB (12)<br>DLB+AD (23)<br>pure AD (89)	Illusions	CUSPAD	VH and illusions were more frequent in pure DLB compared with the other groups<br>*no statistical association with minor visual phenomena*	-

AD – Alzheimer’s Disease, aMCI – amnestic mild cognitive impairment, BPP – Bistable Percept Paradigm, CGI-S – Clinical Global Impression-Severity scale, CN – cognitively normal, CVH – complex visual hallucinations, CUSPAD – Columbia University Scale for Psychopathology in Alzheimer's Disease, DLB – Dementia with Lewy Bodies, ED – eye disease, FOP – feeling of presence, HC – healthy controls, iRBD – idiopathic rapid-eye-movement sleep behavior disorder, LB – Lewy Bodies, LBD – Lewy Body Disease, MCI – mild cognitive impairment, MDS-UPDRS – unified Parkinson's disease rating scale, MVP – minor visual phenomena, MVH – minor visual hallucinations including only illusions, feeling of passage and presence, NEVHI – North-East Visual Hallucinations Interview, NPI – Neuropsychiatric Inventory, NPS – Neuropsychological assessment, PCA – Posterior cortical atrophy, PD – Parkinson’s Disease, PDD – Parkinson’s Disease Dementia, PsycH-Q – Psychosis and Hallucinations Questionnaire, ROC – Receiver operating characteristic, QPE – Questionnaire for Psychotic Experiences, SCOPA-PC – SCales for Outcomes in PArkinson's disease-Psychiatric Complications, VH – visual hallucinations.

Supplementary Table S2 Summary of the characteristics, neuropsychological assessment, and statistical associations between minor visual phenomena and visuoperceptual/visuospatial abilities of the 44 studies included in the systematic review

**Authors (year)**	**Sample (N)**	**Phenomenon**	**NPS assessment**	**Associations with visuoperceptual/spatial deficits**	**Statistical Analysis**
Alsemari and Boscarino (2024) [37]	DLB (94) DLB-MCI (97) HC (56)	Pareidolias	MoCA, digit span forward and backward, Stroop test, Benson Complex Figure test	Dementia severity, Digit Span, Stroop, Benson Figure Copy tests explain 23.8% of the variance in NPT score	Hierarchical multiple linear regression analysis
Chiba et al. (2015) [43]	DLB-AD+ (12), DLB-AD- (11) AD (10) HC (11)	FOP	WAIS-III, WMS-R, Bender-Gestalt Test	DLB-AD+ displayed higher scores on Bender-Gestalt test compared to HC, DLB-AD-, AD *no statistical association with minor visual phenomena*	-
D'Antonio et al. (2022) [15]	35 LBD (19 DLB and16 PDD)	MVH (illusions, feeling of presence, feeling of passage)	RAVLT, Corsi Block, Digit Span, Babcock Story Recall Test, Rey-Osterrieth Complex Figure Test, the Visual Search Test, TMT, the Clock Drawing Test, Copy of the Rey-Osterrieth Complex Figure Test, Semantic and Phonemic Verbal Fluency Test, Boston Naming Test, Raven's Colored Progressive Matrices, FAB, Benton Line	No significant association between MVH and visuoperceptual/spatial tests performance	Spearman correlation

			test, VOSP, the "Length Match Task", "Size Match Task", "Orientation Match Task"; "Position of Gap Match Task" subtests of the BORB		
D'Antonio et al. (2024) [17]	28 LBD with history of VH (16 DLB, 12 PDD)<br>HC (20)	MVH (illusions, FOP, feeling of passage)	Not tested	Not tested	-
Ferman et al. (2013) [22]	LBD (41)<br>AD (70)<br>AD amy-LB (14)	Misperceptions	Not tested	Not tested	-
Firbank et al. (2024) [44]	LBD-VH (41)<br>LBD-noVH (48)<br>HC (60)	Pareidolias, MVH (illusions, feeling of presence, feeling of passage)	ACE-R (total and visuospatial score) and CAMCOG (total and praxis score), a visual angle discrimination test	Not tested	-
Galvin et al. (2021) [45]	DLB (110)<br>AD (78)<br>HC (53)<br>MCI DLB (22)<br>MCI AD (79)	Pareidolias	UDSv3.0, animal naming, numbers forward, numbers backward, TMT A, TMT B, MINT, HVLT immediate, delay, recognition	Not tested	-
Hamilton et al. (2021) [46]	probable MCI-LB (43)	Pareidolias	ACE-R, NART, CDR, MMSE	Not tested	-

	possible MCI-LB (20)<br>MCI-AD (40)<br>HC (34)				
Heitz et al. (2015) [47]	DLB VH (36)<br>DLB no VH (30)	Illusions	FAB, FCSRT, TMT, digit span forward and backward, lexical evocation formal and semantic, Rey-Osterrieth complex figure	Subgroup comparisons based on the type of hallucinations (complex, illusions, simple) no significant differences between illusion group and the other two groups	Kruskal-Wallis test
Hely et al. (1996) [48]	DLB (9)	Illusions	Two patients underwent a complete neuropsychological assessment (test not specified)	At baseline, patient with illusions had also slight visual memory impairment, which declined over time.<br>*no statistical association with the minor visual phenomena*	-
Inagawa et al. (2020) [49]	probable DLB (24) AD (22)	Pareidolias	MMSE	Not tested	-
Iseki et al. (2002) [20]	probable DLB (8)	Illusions, FOP	MMSE, WAIS-R, Raven Colored Matrices	Not tested	-
Ishimaru et al. (2024) [50]	DLB (2)	Illusions	MMSE, ACE-III	Not tested	-
Mamiya et al. (2016) [12]	probable DLB (52)<br>AD (52)	Pareidolias	ACE-R, Shape detection screening and Position Discrimination (subtests	Significant negative correlation between scene pareidolia test score and visuospatial score of the ACE-R.	Pearson's correlation

	HC (20)		VOSP), Face recognition subtest (Visual Perception Test for Agnosia)		
Matar et al. (2020) [51]	DLB (27)<br>HC (25)	Misperception	TMT A-B, RAVLT, clock drawing test, Boston naming, verbal fluency, forward Digit Span	Not tested	-
McCann et al. (2023) [33]	DLB-PDD (13)<br>PD (13)<br>AD (12)<br>PCA (5)<br>HC (32)	Pareidolias	ACE-III, Rey-Osterrieth Complex Figure (copy, recall), cube analysis, incomplete letters, progressive silhouettes (VOSP), RAVLT, Naming, Digit Span forward and backward	Significant negative correlation between composite visuoperception score and pareidolic responses. Visuoperception score significant predictor of pareidolic responses (negative association).	Spearman's correlation<br>Linear regression
Morenas-Rodríguez et al. (2018) [52]	Probable DLB (81)	FOP and feeling of passage	Digit Span Forward and Backward, TMT-A-B, FCSRT (free recall, total recall, delayed free recall and delayed total recall), Boston naming test, Rey-Osterrieth complex figure test-copy, number localization (VOSP subtest)	No significant group differences found for visuospatial and visuoperceptual tests.	K-means clustering method- t-test-Mann-Whitney U test
Mori et al. (2020) [53]	probable DLB (24)<br>probable AD (48)	FOP	CDR, MMSE, visual perception test (discrimination of size and form, overlapping figure identification, visual	No significant difference in the visuoperceptual task scores between those with and those without FOP.	Mann-Whitney test group differences

			counting tasks), block design subtest of WAIS-R		
Moylett et al. (2019) [54]	DLB (251)	Illusions	MMSE	Not tested	-
Nagahama et al. (2007) [30]	DLB (100)	FOP	MMSE	Not tested	-
Nakata et al. (2022) [55]	DLB (147)<br>MCI DLB (15)	Pareidolias	MMSE and visual/visuospatial assessment (tests not specified)	Not tested	-
Nicastro et al. (2020) [19]	DLB (25)	FOP	MMSE	Not tested	-
Oishi et al. (2020) [56]	DLB (37)<br>AD (58)<br>HC (32)	Pareidolias	CDR, MMSE, Digit span forward and backward, FAB, ADAS, VOSP (shape detection, cube analysis), Visual material identification tests (visual texture recognition), the position in space subtest from the Developmental Test of Visual Perception	Significant positive correlation between object recognition test scores (in non-canonical blurred and clear texture conditions) and material identification test, cube analysis, and position in space scores.<br>Significant negative correlation between number of pareidolia-like responses and material identification score.<br>Significant lower score in real material identification score in DLB	Spearman's correlation<br>One-way ANOVA group-difference

				patients with pareidolia-like responses than in those without them.	
Phillips et al. (2021) [57]	DLB (23)<br>HC (20)	Misperceptions	MMSE, Rey Figure Copy, Clock drawing test, TMT A	No significant association between Rey Figure Copy and misperceptions.	Spearman's correlation
Phillips et al. (2022) [23]	iRBD-MCI probable prodromal DLB (4)	Misperceptions	SART, Mental rotation, visuo-spatial task, MoCA, WMS-r (logical memory 1 and 2), RAVLT, Digit span forward and backward, TMT, clock, Stroop, phonemic fluency, semantic fluency (animals)	Not tested	-
Posner et al. (2001) [58]	DLB (1)	Illusions	Formal neuropsychological testing<br>(test not specified)	Patient had visuospatial skills impaired<br>*no statistical association with the minor visual phenomena*	-
Rahman-Filipiak et al (2022) [59]	LBD (56)<br>AD (44)<br>aMCI (96)<br>non-a MCI (61)<br>HC (202)	Pareidolias	Speeded Attention Task, TMT, MINT, Benton Copy	Significant positive relationships between Noise pareidolia faces correct and Benton Copy.<br>Significant negative relationships between pareidolic errors and Benton Copy.	Partial correlation (discriminant and convergent validity)
Reckner et al. (2020) [32]	probable DLB (7)<br>PD (18)<br>*with FOP: 25*<br>PD without FOP (25)	FOP	NART, WAIS-III, Recognition memory test, the Camden memory test, Graded naming test, VOSP, phonemic and category	Patients with FOP showed more frequent impairments in visual processing.	Chi-squared group comparison

			fluency, Color Word test, Hayling, Digit Symbol Coding and Symbol search from the WAIS-III, SDMT		
Revankar et al. (2024) [60]	DLB (25)<br>AD (29)<br>PD (5)<br>HC (11)	Pareidolias	MMSE-J (japanese version)	Not tested	-
Rothenberg et al. (2023) [61]	DLB (4)	Illusions	MOCA	Not tested	-
Stavitsky et al. (2006) [62]	DLB (28)<br>AD (55)	Illusions	modified MMSE (mMMS), HVLT-Revised	DLB patients compared with AD showed visual illusions and more impairment in the visuocostructional domain<br>*no statistical association with minor visual phenomena*	-
Suárez-González et al. (2014) [25]	probable DLB (80)<br>probable AD (85)	Illusions, FOP	MMSE	Not tested	-
Sumi et al. (2022) [18]	DLB (3)<br>*converted from a sample of iRBD (36)*	FOP, feeling of passage, illusions, pareidolias	MMSE, FAB, MoCA, BACS	Not tested	-
Suzuki et al. (2017) [63]	DLB (8)<br>HC (9)	Pareidolias	MMSE	Not tested	-

Taomoto et al. (2022) [64]	Prodromal DLB (2)	Illusions	MMSE	Not tested	-
Taomoto et al. (2024) [65]	Prodromal DLB (5)	Illusion, feeling of passage, FOP	MMSE	Not tested	-
Uchiyama et al. (2012) [27]	probable DLB (34)<br>AD (34)<br>HC (26)	pareidolias	Digit span, object naming, shape detection and position discrimination (subtests VOSP), object decision, face recognition	Significant negative correlations between illusory responses and face recognition (only in those taking donepezil).<br>Trend between illusory responses and position discrimination (only in those taking donepezil).<br>No significant associations for the entire DLB sample and those without donepezil.	Pearson's correlation
Urwyler et al. (2016) [66]	LBD (79)*<br>ED (135),<br>PD (156)<br>HC (164)<br>*among which PDD (48) and DLB (31)	Illusions, FOP and feeling of passage (combined)	MMSE, Verbal and categorical fluency	Not tested	-
van de Beek et al. (2021) [67]	Total DLB (100)<br>Probable DLB (73)<br>MCI (27)	MVH (illusions, passage, presence)	VAT, immediate recall and delayed recall of the Dutch version of the verbal learning test,	Not tested	-

			TMT-A-B, Stroop test, Digit Span forward and backward, letter fluency, FAB, number-location test, dot counting, and fragmented letters (VOSP subtests), VAT naming and category fluency.		
Watanabe et al. (2018) [68]	DLB (36) AD (12)	Pareidolias	MMSE, ACE-R, shape detection and position discrimination (VOSP subtests), face recognition of Visual perception Test for Agnosia	Not tested	-
Watanabe et al. (2023) [36]	DLB (43)	Pareidolias	Digit Span, Total score on the visual processing test (sum of VPTA face recognition, VOSP shape detection, VOSP position discrimination)	Factor 3 (visual processing) weighted strongly on higher visual processing test scores and lower pareidolia responses.	Factor analysis
Watanabe et al. (2020) [69]	Probable DLB (1)	Pareidolias	WAIS-III, WMS-R, ACE-R, Digit span, Spatial span, WAB, Token.	Visuospatial abilities were normal across visits *no statistical association with minor visual phenomena*	-
Yokoi et al. (2014) [26]	probable DLB (34) AD (34) HC (28)	Pareidolias	Digit span, spatial span, shape detection and position discrimination (subtests VOSP), object	Significant negative correlations between illusory responses (face version) and shape detection, face recognition, spatial span. significant negative correlations between	Pearson's correlation

			decision, face recognition, FAB, verbal fluency	illusory responses (object version) and shape detection and spatial span.	
Yoshizawa et al. (2013) [70]	pure DLB (12) DLB+AD (23) pure AD (89)	Illusions	SRT, the 15 item Boston Naming Test, verbal fluency tests for letter (CFL) and category (animal), the five item Rosen Drawing Test, and the Digit Span	Higher visuospatial impairment in pure DLB compared with the other groups *no statistical association with minor visual phenomena*	-

ACE-R – Addenbrooke's Cognitive Examination Revised, AD – Alzheimer's Disease, ADAS – Alzheimer's Disease Assessment Scale, aMCI – amnestic mild cognitive impairment, BACS – Brief Assessment of Cognition in Schizophrenia, BORB – Birmingham Object Recognition Battery, BPP – Bistable Percept Paradigm, CAMCOG – Cambridge Cognition Examination, CDR – Clinical Dementia Rating, CN – cognitively normal, CVH – complex visual hallucinations, DLB – Dementia with Lewy Bodies, ED – eye disease, FAB – Frontal Assessment Battery, FCSRT – Free and Cued Selective Reminding Test, FOP – feeling of presence, HC – healthy controls, HVLT – Hopkins Verbal Learning Test, iRBD – idiopathic rapid-eye-movement sleep behavior disorder, LB – Lewy Bodies, LBD – Lewy Body Disease, MCI – mild cognitive impairment, MINT – Multilingual Naming Test, MMSE – Mini-Mental State Examination, MVP – minor visual phenomena, MVH – minor visual hallucinations including only illusions, feeling of passage and presence, NART – National Adult Reading Test, NEVHI – North-East Visual Hallucinations Interview, NPS – Neuropsychological assessment, PCA – Posterior cortical atrophy, PD – Parkinson's Disease, PDD – Parkinson's Disease Dementia, RAVLT – Rey Auditory Verbal Learning Test, SART – Sustained Attention Response Task, SDMT – Symbol Digit Modalities Test, SRT – Selective Reminding Test, TMT – Trail Making Test, UDSv3.0 – Uniform Data Set version 3.0, VAT – Visual association test, VOSP – Visual Object and Space Perception Battery, VPTA – Visual Perception Test for Agnosia, WAB – Western Aphasia Battery, WAIS-III – Wechsler Adult Intelligence Scale, WMS-R – Wechsler Memory Scale—Revised.

Supplementary Table S3 Quality assessment for all quantitative studies (n = 36) using the Effective Public Health Practice Project Quality Assessment Tool for Quantitative Studies (EPHPP)

Authors (year)	A) Selection bias				B) Study design			C) Confounders				D) Blinding				E) Data collection				F) Withdrawals and Drop-out				Global Index	Global Rating
	Q1	Q2	SC	R	TS	SC	R	Q1	Q2	SC	R	Q1	Q2	SC	R	Q1	Q2	SC	R	Q1	Q2	SC	R		
Alsemari and Boscarino (2024) [37]	1	5	2	m	4	2	m	1	1	1	s	1	2	2	m	1	1	1	s	4	5	2	m	1	s
Chiba et al. (2015) [43]	2	1	2	m	4	2	m	1	1	1	s	1	1	3	w	2	2	3	w	4	5	2	m	3	w
D'Antonio et al. (2022) [15]	1	1	1	s	7	3	w	2		1	s	1	2	2	m	1	1	1	s	1	1	1	s	2	m
D'Antonio et al. (2024) [17]	2	1	2	m	4	2	m	1	1	1	s	1	1	3	w	1	1	1	s	4	5	2	m	2	m
Ferman et al. (2013) [22]	1	1	1	s	5	2	m	1	4	3	w	1	2	2	m	3	3	3	w	4	5	2	m	3	w
Firbank et al. (2024) [44]	1	1	1	s	4	2	m	1	2	2	m	1	2	2	m	1	1	1	s	4	5	2	m	1	s
Galvin et al. (2021) [45]	2	5	2	m	4	2	m	1	3	3	w	1	1	3	w	1	1	1	s	4	5	2	m	3	w
Hamilton et al. (2021) [46]	1	2	2	m	5	2	m	1	1	1	s	1	1	3	w	1	1	1	s	2	4	3	w	3	w
Heitz et al. (2015) [47]	2	2	2	m	4	2	m	2		1	s	1	2	2	m	3	3	3	w	4	5	2	m	2	m
Inagawa et al. (2020) [49]	1	1	1	s	7	2	m	1	1	1	s	1	1	3	w	1	1	1	s	4	5	2	m	2	m
Mamiya et al. (2016) [12]	2	1	2	m	7	3	w	2		1	s	1	1	3	w	1	1	1	s	4	5	2	m	3	w
Matar et al. (2020) [51]	1	1	1	s	4	2	m	1	1	1	s	1	1	3	w	1	1	1	s	4	5	2	m	2	m

McCann et al. (2023) [33]	1	5	2	m	5	2	m	1	1	1	s	1	3	3	w	1	1	1	s	4	5	2	m	2	m
Morenas-Rodríguez et al. (2018) [52]	2	5	2	m	7	3	w	3	3	3	w	1	1	3	w	3	3	3	w	4	5	2	m	3	w
Mori et al. (2020) [53]	1	1	1	s	4	2	m	1	1	1	s	1	1	3	w	3	3	3	w	4	5	2	m	3	w
Moylett et al. (2019) [54]	2	5	2	m	5	2	m	1	3	3	w	1	2	2	m	3	3	3	w	4	5	2	m	3	w
Nagahama et al. (2007) [30]	2	5	2	m	7	3	w	1	4	3	w	1	1	3	w	3	3	3	w	4	5	2	m	3	w
Nakata et al. (2022) [55]	2	2	2	m	7	3	w	1	3	3	w	1	1	3	w	1	1	1	s	4	5	2	m	3	w
Nicastro et al. (2020) [19]	2	5	2	m	4	2	m	2		1	s	1	2	2	m	2	2	3	w	4	5	2	m	3	w
Oishi et al. (2020) [56]	1	1	1	s	4	2	m	2		1	s	1	1	3	w	3	3	3	w	4	5	2	m	3	w
Phillips et al. (2021) [57]	1	1	1	s	4	2	m	1	1	1	s	1	1	3	w	1	2	2	m	4	5	2	m	2	m
Phillips et al. (2022) [23]	2	1	2	m	5	2	m	1	1	1	s	1	1	3	w	1	1	1	s	1	3	3	w	3	w
Rahman-Filipiak et al (2022) [59]	1	1	1	s	7	3	w	1	3	3	w	1	1	3	w	1	1	1	s	4	5	2	m	3	w
Reckner et al. (2020) [32]	1	5	2	m	5	2	m	2		1	s	1	2	2	m	3	3	3	w	4	5	2	m	2	m
Revankar et al. (2024) [60]	1	1	1	s	7	3	w	3		3	w	1	1	3	w	1	1	1	s	4	5	2	m	3	w
Stavitsky et al. (2006) [62]	2	1	2	m	5	2	m	1	1	1	s	1	1	3	w	1	1	1	s	1	1	1	s	2	m

Suárez-González et al. (2014) [25]	2	1	2	m	7	3	w	2		1	s	1	1	3	w	1	1	1	s	4	5	2	m	3	w
Sumi et al. (2022) [18]	2	5	2	m	5	2	m	1	1	1	s	1	2	2	m	1	1	1	s	1	1	1	s	1	s
Suzuki et al. (2017) [63]	2	1	2	m	4	2	m	1	1	1	s	1	1	3	w	1	1	1	s	4	5	2	m	2	m
Uchiyama et al. (2012) [27]	2	1	2	m	5	2	m	2		1	s	1	1	3	w	1	1	1	s	4	5	2	m	2	m
Urwyler et al. (2016) [66]	2	1	2	m	4	2	m	1	3	3	w	1	2	2	m	1	1	1	s	4	5	2	m	2	m
van de Beek et al. (2021) [67]	1	1	1	s	5	2	m	1	4	3	w	1	1	3	w	1	1	1	s	1	1	1	s	2	m
Watanabe et al. (2018) [68]	1	1	1	s	4	2	m	2		1	s	1	1	3	w	1	1	1	s	4	5	2	m	2	m
Watanabe et al. (2023) [36]	2	1	2	m	7	3	w	3	4	3	w	1	1	3	w	1	1	1	s	4	5	2	m	3	w
Yokoi et al. (2014) [26]	2	5	2	m	4	2	m	1	1	1	s	1	3	3	w	1	1	1	s	4	5	2	m	2	m
Yoshizawa et al. (2013) [70]	1	3	2	m	5	2	m	1	1	1	s	1	1	3	w	1	1	1	s	4	5	2	m	2	m

The quality of the studies included was assessed independently by two reviewers (EC and AQ) using EPHPP [41], (https://www.ephpp.ca/quality-assessment-tool-for-quantitative-studies/), following the component (A-F) ratings that contribute to the global rating for each paper. SC = Score, RT = Rating, TS = type study, w = weak, m = moderate, s = strong.

Supplementary Table S4 Quality assessment for all case reports (n = 7) using the "CARE criteria checklist"

**Authors (years)**	**T**	**KW**	**Abstract**				**Introd**	**Patient Info**				**Clinical Finding**	**Tm**	**Diagnostic Assess**				**Therap Interv**			**Follow-up and Outcomes**				**Discussion**				**PP**	**Cons**
			**a**	**b**	**c**	**d**		**a**	**b**	**c**	**d**			**a**	**b**	**c**	**d**	**a**	**b**	**c**	**a**	**b**	**c**	**d**	**a**	**b**	**c**	**d**		
Hely et al. (1996) [48]	Y	Y	Y	Y	Y	Y	Y	Y	Y	Y	Y	Y	Y	Y	N	Y	N	Y	N	N	Y	N	N	N	N	Y	Y	Y	N	Y
Iseki et al. (2002) [20]	N	Y	N	Y	Y	Y	N	Y	Y	Y	Y	Y	Y	Y	N	N	N	Y	Y	N	N	N	N	N	Y	Y	Y	Y	N	Y
Ishimaru et al. (2024) [50]	Y	Y	Y	Y	Y	Y	Y	Y	Y	Y	Y	Y	Y	Y	N	N	N	Y	Y	N	Y	Y	N	N	Y	Y	Y	Y	N	Y
Posner et al. (2001) [58]	N	N	N	Y	Y	N	N	Y	Y	Y	Y	Y	Y	Y	N	Y	N	Y	Y	Y	Y	N	Y	N	N	N	Y	N	N	N
Rothenberg et al. (2023) [61]	Y	Y	Y	Y	Y	Y	Y	Y	Y	Y	Y	Y	Y	Y	N	Y	N	Y	Y	Y	Y	Y	Y	N	Y	Y	Y	Y	N	N
Taomoto et al. (2022) [64]	Y	Y	N	Y	Y	Y	N	Y	Y	Y	Y	Y	Y	Y	N	Y	N	Y	Y	Y	Y	Y	N	N	Y	Y	Y	Y	N	Y
Taomoto et al. (2024) [65]	Y	Y	N	Y	Y	Y	N	Y	Y	Y	Y	Y	Y	Y	N	Y	N	Y	Y	N	Y	Y	N	N	Y	Y	Y	Y	N	Y

The quality of the studies included was assessed independently by two reviewers (EC and AQ) using CARE [40]. T = Title, KW = Keywords, Introd = Introduction, Tm = Timeline, Diagnostic Assess = Diagnostic assessment, Therap Interv = Therapeutic Intervention, PP = Patient Perspective, Cons = Consent, Y = yes, N = no.
